# Behavioral alterations in long-term *Toxoplasma gondii* infection of C57BL/6 mice are associated with neuroinflammation and disruption of the blood brain barrier

**DOI:** 10.1371/journal.pone.0258199

**Published:** 2021-10-05

**Authors:** Leda Castaño Barrios, Ana Paula Da Silva Pinheiro, Daniel Gibaldi, Andrea Alice Silva, Patrícia Machado Rodrigues e Silva, Ester Roffê, Helton da Costa Santiago, Ricardo Tostes Gazzinelli, José Roberto Mineo, Neide Maria Silva, Joseli Lannes-Vieira

**Affiliations:** 1 Laboratory of Biology of the Interactions, Oswaldo Cruz Institute/Fiocruz, Rio de Janeiro, Rio de Janeiro, Brazil; 2 Multiuser Laboratory for Research Support in Nephrology and Medical Sciences, Federal University Fluminense, Niterói, Rio de Janeiro, Brazil; 3 Laboratory of Inflammation, Oswaldo Cruz Institute/Fiocruz, Rio de Janeiro, Rio de Janeiro, Brazil; 4 Laboratory of Molecular Immunology, National Institute of Allergy and Infectious Diseases, National Institutes of Health, Bethesda, Maryland, United States of America; 5 Department of Biochemistry and Immunology, Federal University of Minas Gerais, Belo Horizonte, Minas Gerais, Brazil; 6 Institute of Biomedical Sciences, Federal University of Uberlândia, Uberlândia, Minas Gerais, Brazil; Lewis Katz School of Medicine, Temple University, UNITED STATES

## Abstract

The Apicomplexa protozoan *Toxoplasma gondii* is a mandatory intracellular parasite and the causative agent of toxoplasmosis. This illness is of medical importance due to its high prevalence worldwide and may cause neurological alterations in immunocompromised persons. In chronically infected immunocompetent individuals, this parasite forms tissue cysts mainly in the brain. In addition, *T*. *gondii* infection has been related to mental illnesses such as schizophrenia, bipolar disorder, depression, obsessive-compulsive disorder, as well as mood, personality, and other behavioral changes. In the present study, we evaluated the kinetics of behavioral alterations in a model of chronic infection, assessing anxiety, depression and exploratory behavior, and their relationship with neuroinflammation and parasite cysts in brain tissue areas, blood-brain-barrier (BBB) integrity, and cytokine status in the brain and serum. Adult female C57BL/6 mice were infected by gavage with 5 cysts of the ME-49 type II *T*. *gondii* strain, and analyzed as independent groups at 30, 60 and 90 days postinfection (dpi). Anxiety, depressive-like behavior, and hyperactivity were detected in the early (30 dpi) and long-term (60 and 90 dpi) chronic *T*. *gondii* infection, in a direct association with the presence of parasite cysts and neuroinflammation, independently of the brain tissue areas, and linked to BBB disruption. These behavioral alterations paralleled the upregulation of expression of tumor necrosis factor (TNF) and CC-chemokines (CCL2/MCP-1, CCL3/MIP-1α, CCL4/MIP-1β and CCL5/RANTES) in the brain tissue. In addition, increased levels of interferon-gamma (IFNγ), TNF and CCL2/MCP-1 were detected in the peripheral blood, at 30 and 60 dpi. Our data suggest that the persistence of parasite cysts induces sustained neuroinflammation, and BBB disruption, thus allowing leakage of cytokines of circulating plasma into the brain tissue. Therefore, all these factors may contribute to behavioral changes (anxiety, depressive-like behavior, and hyperactivity) in chronic *T*. *gondii* infection.

## 1. Introduction

The protozoan parasite *Toxoplasma gondii* is the etiologic agent of toxoplasmosis [1, 2]. Currently, this infection afflicts a third of the world population, with a seroprevalence ranging from 0.8 to 92%, depending on the region and habits of the populations [3, 4]. For instance, in Brazil and USA the seroprevalence is of 92% and 22.5%, respectively [4, 5]. The parasite may invade any cell type and infect all body tissues showing tropism for the central nervous system (CNS) [3, 6]. The acute phase of *T*. *gondii* infection is characterized by presence of tachyzoite forms and the chronic phase is defined by the presence of encysted bradyzoite forms, called tissue cysts [7]. The drugs currently available for etiological treatment of *T*. *gondii* infection are more effective in the acute phase, however they do not offer a parasitological cure in the chronic phase [8]. Thus, the cysts cannot be eliminated, remaining in the CNS throughout the life of the host, apparently in silent state [7] and with sustained neuroinflammation during the acute and chronic phase in mouse models of infection [9, 10]. In humans, neuroinflammation has been linked to the development of neurodegenerative illnesses also associated with behavioral alterations as Alzheimer disease [11]. Several studies have investigated the role of *T*. *gondii* infection as a risk factor for neurological and behavioral disorders. In humans, infection by *T*. *gondii* has been related to mental illnesses such as schizophrenia, bipolar disorder, depression and obsessive-compulsive disorder, as well as mood, personality and other behavioral changes [12], as increased rate of involvement in traffic accidents [13], suicide attempts [14] and alteration of cognitive functioning [15]. Alike humans, mice are intermediate hosts of *T*. *gondii* [16] and show behavioral abnormalities. In experimentally infected mice, among the reported behavioral changes are loss of predator fear [17, 18], decreased anxiety [10], increased exploratory behavior [19, 20] and impairment of long-term memory during chronic infection [21]. All these findings contributed to the hypothesis of host manipulation by the parasite, contributing to exposition to definitive host predator and *T*. *gondii* cycle maintenance.

Behavioral alterations have been proposed to be independent of persistent neuroinflammation [18] and the apparent presence of *T*. *gondii* cysts [18, 22] in the CNS. In non-infectious and infectious experimental models, behavioral alterations have been linked to neuroinflammation or independent of it [23–25]. Further, systemic inflammatory profile associated with increased circulating cytokine levels raised as a contributor to underpin behavioral alterations in infectious diseases as hepatitis and Chagas disease [24, 26]. Therefore, in the present work we carried out a kinetics study of infection of C57BL/6 mice with the ME-49 type II strain of *T*. *gondii* to settle initially a model of long-term infection to study behavioral changes. For that, we used standardized tests to evaluate the presence of anxiety, depressive-like behavior, and hyperactivity. Further, trying to shed light on the biological factors associated with infection-induced behavioral abnormalities, we assessed cyst numbers and presence of neuroinflammation as well as their topographical localization in the CNS areas, cytokine expression in the brain tissue and serum, and BBB integrity.

## 2. Materials and methods

### 2.1 Ethics statement

The experimental procedures were performed in accordance with the recommendations of the Guide for the Care and Use of Laboratory Animals of the National Council for Animal Experimentation. The Animal Use Ethics Committee of Oswaldo Cruz Institute/Fiocruz approved all procedures performed in this study (license L014/2018). All the data presented were obtained from two independent experiments registered in the Experience Record Book #73, LBI/IOC-Fiocruz.

### 2.2 Experimental design

**Fig 1:**
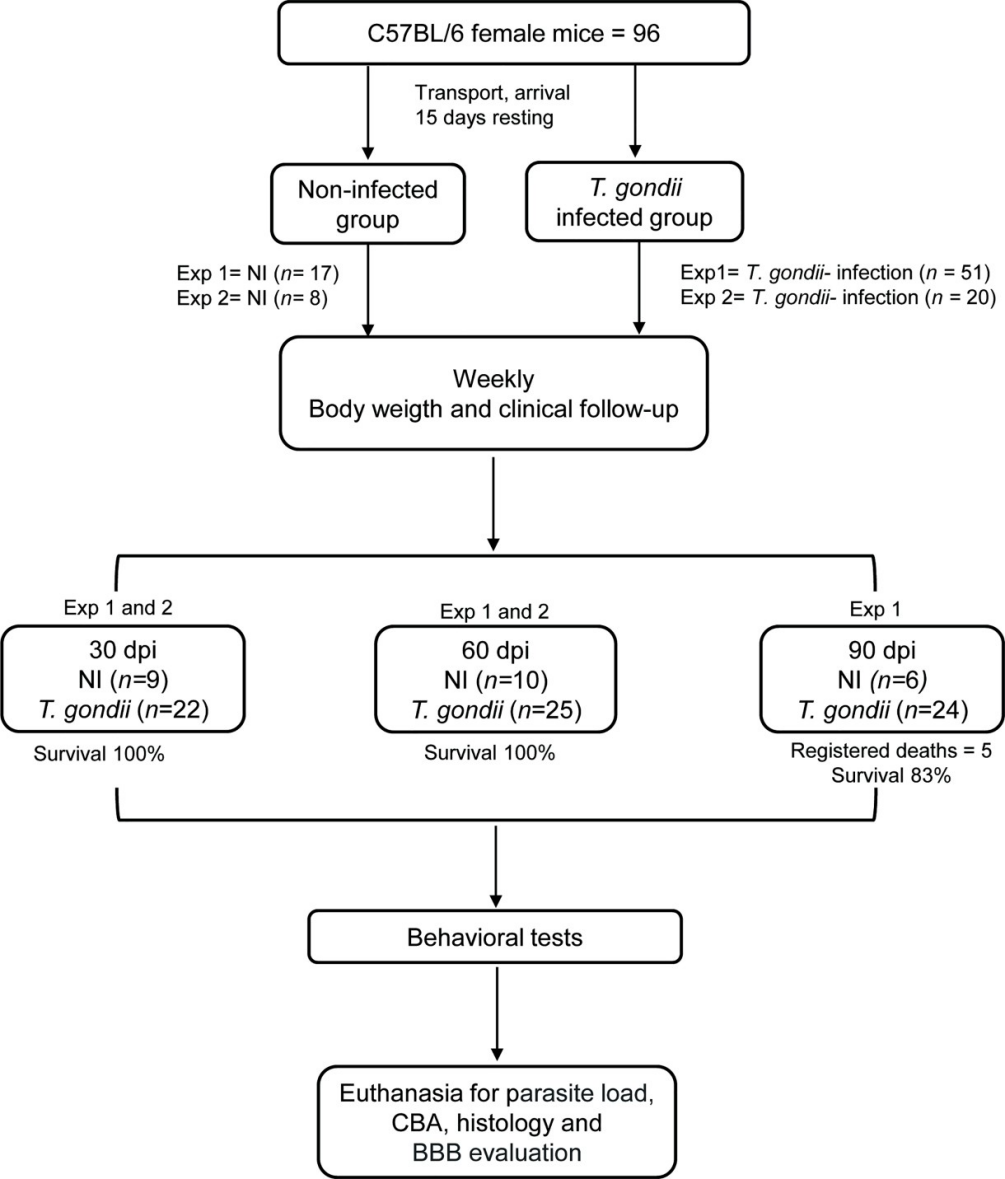
Flow chart showing the experimental protocol with the number of animals used. Death registered, and mice included in 2 (endpoint registered at “30 and 60 dpi”) or 1 (endpoint registered at “90 dpi”) independent experiments.

Experimental check list is described in Author´s Check List (S1 Checklist). A total of 116 female mice of the C57BL/6 (H-2^b^) lineage, 3-4-week-old, was provided by the Institute of Science and Technology in Biomodels (ICTB) of Oswaldo Cruz Foundation and housed in the Experimental Animal Facility (CEA-CF/IOC unit) under specific pathogen-free conditions, in polypropylene cages lined with pine sawdust and kept in microisolators, under noise and light controlled conditions (12 hours light/12 hours dark). Animals were randomly grouped into groups of 3–5 mice per cage and received water and grain-based *ad libitum*. To minimize the effects of stress and allow the adaptation process to the new environment, mice were kept without manipulation for 15 days in the cages, provided with environmental enrichment (igloo). After the adaptation period, the animals were infected and analyzed according to the experimental protocols (Fig 1).

At arrival, experimental groups were formed. The cages were numbered and randomly classified for experimental infection and analysis at the indicated timepoints. Experimental groups were formed with a total of 96 mice divided into two replications: Experiment 1 (i) 30 days postinfection (dpi): 5 non-infected (NI) controls and 12 infected; (ii) 60 dpi: 6 NI controls and 15 infected; and (iii) 90 dpi: 6 NI controls and 24 infected. Experiment 2 (i) 30 dpi: 4 NI controls and 10 infected; and (ii) 60 dpi: 4 NI controls and 10 infected. Additionally, 20 mice divided in two independent experiments of 10 mice each were used to study cytokine expression in the CNS at 20 dpi (2 NI, 3 infected) and at 45 dpi (2 NI, 3 infected), respectively, before and after neuroinflammation onset S1 Fig.

### 2.3 *Toxoplasma gondii* infection and clinical follow-up

Animals were infected orally with five cysts of the cystogenic ME-49 *T*. *gondii* strain [27], provided by Dra. Neide Maria da Silva (ICBIM, UFU) and kept in the Laboratory of Biology of the Interactions (LBI-IOC) by serial passages in C57BL/6 (H-2^b^) female mice every 60 days. The clinical follow-up was carried out weekly, registering the following parameters: piloerection, apathy, prostration, mobility, posture, aggressive behavior, pain, mortality, and weight loss, assessed using a mouse precision scale (Sartorius ED623S Milligram Scale, OCE, USA) and used as an indicator of hyporexia. Signs of pain or suffering such as isolation from the group, loss of body weight greater than 30% of the initial weight, injuries from fights, ataxia, immobility, and any death outside of planned euthanasia or humane endpoints were the criteria established to guide the decision-making endpoint.

### 2.4 Behavioral tests

All behavioral tests were performed between 8:00 am and 4:00 pm and recorded on a DSC-DVD810 video camera (Sony, USA). To minimize stress and increase familiarity, all behavioral tests applied to the different experimental groups were performed in an environment provided with 12 hours of light and 12 hours of dark cycle at a temperature of 22 ± 2°C and a noise level of approximately 40 dB produced by an air conditioner. The experimental groups (30, 60 and 90 dpi) were subjected to behavioral tests, no mouse was subjected to the same test more than once, but animals were reused in different tests to reduce the number of mice used in the research. Behavioral tests were performed from the least stressful to the most stressful: (i) open field test (OFT), (ii) grip strength meter test (GSMT) (iii) tail suspension test (TST), (iv) forced-swimming test (FST) and (v) footprint test [24]. After testing each mouse, the device was cleaned with 70% alcohol.

### 2.5 Open Field Test (OFT)

To assess anxiety, we used the OFT based on the exploratory profile of rodents denominated as thigmotaxis. Once exposed to a new environment, they will prefer to be close to the wall or peripheral areas, instead of being exposed in the central area of the field, which represents danger as it is the most exposed area. As time passes, anxiety levels decrease due to habituation and the mouse ventures to explore the central area, a shorter distance traveled in the central area. The less time exploring that area is an indication of anxious behavior [28, 29]. The open field test consists of a 60 cm acrylic cubic box, with white walls and soil divided by black lines into 49 equal squares, where the animal is exposed to an environment without aversive or rewarding stimuli. The mouse was allowed to freely explore the open field for 5 minutes. OFT is used to assess exploratory activity and/or locomotion; anxiety was evaluated as time expend in the central zone (s); the distance traveled (cm) was calculated by the number of lines crossed during the total time; speed or velocity was estimated as time spent crossing the lines (cm/s); and immobility time (s) in total time.

### 2.6 Grip strength meter test (GSMT)

The grip strength meter apparatus (EFF 305, Insight, Brazil) was used to the GMST, a non-invasive method to assess the strength of the muscle of mice limbs [30]. It is composed of a metal bar fixed to a force transducer that measures the peak of traction (in gram-force) displayed on a digital screen. The test is based on the natural tendency of mice to grab a horizontal metal bar when slightly pulled by the tail for 2–3 seconds, consisting of three consecutive repetitions in 15s. Data are shown as mean of strength intensity = gram-force (gf)/body weight (g).

### 2.7 Tail suspension test (TST)

The test is based on the principle that mice placed in an unavoidable but moderately stressful situation will develop a motionless posture, indicative of depression-like behavior [31]. The device, called tail suspension apparatus (Insight, Brazil), consists of a large box measuring 61.10 cm wide by 55.40 cm high, divided into four identical 15.28 cm shares, allowing testing four animals at a time, and a bar of aluminum suspension placed horizontally at the top, where the animal hangs by the tail with the help of a ribbon. The division of the shares are arranged in such a way that, when the mice are hung, they cannot touch the other walls of the compartment or observe the others. The C57BL/6 strain has the ability to reach and climb using its own tail [32], thus we employed hollow transparent polycarbonate cylinders (4 cm long, 1.6 cm outside diameter, 1.3 cm inside diameter, 1.5 grams), placed around the tails to prevent climbing behavior. Shaking, reaching out, swinging vigorously, and body torsion or jerky are considered active movements. As the mice start to tire, the movements become more subtle until only the front legs move, which is considered immobility, as well as the balance of the body resulting from previous movements, passive oscillations, and total absence of movements. The test was recorded with a video camera (Sony, USA) for 5 minutes, and the total duration of immobility in 5 minutes was registered.

### 2.8 Forced swimming test (FST)

This test was used to evaluate depressive-liked behavior. The test consists of a cylinder (height 35 cm, diameter 25 cm) containing clean water (25 ± 1°C), up to a level of 20 cm above the bottom, where the mice were gently placed on the surface. The test lasted a total of 6 min, the first 2 minutes was considered habituation and the total duration of immobility was recorded during the last 4 minutes [33]. Active movements were defined as the time during which the mouse made strong movements against the cylinder walls using the forelimbs. Immobility was defined as the time during which the mouse remained floating passively, and made no attempt to escape, thus showing only slow movements to keep its head above water. The water was changed before the introduction of each animal. After test, the animal was dried with gauze and replaced in its cage.

### 2.9 Footprint

The mouse gait analysis was performed using the footprint test. The paws were covered with non-toxic ink (red color to paint the forelimbs; blue color to the hindlimbs). The mouse was free to walk on a sheet of white paper (12 cm wide; 29.7 cm length) to generate a footprint pattern [34]. The following parameters were evaluated: stride length of the anterior and posterior limbs, width of the front and rear base, overlapping distance between the anterior and posterior limbs and the spread of the fingers [35]. For analysis, we used the free software Image J (NIH). In order to generate reliable scoring data, footprints with 4–6 consecutive steps from each foot were analyzed. For each step parameter, three values were measured from each animal footprint, excluding footprints made at the beginning and at the end of the run where the animal was initiating and finishing movement, respectively. The mean value of each set of three values was used in subsequent analysis. The data are presented as the mean of the analyzed parameter considering the body weight as the correct factor = parameter (cm)/body weight (g), as previously described [36].

### 2.10 Determination of blood-brain barrier integrity, obtention of brain tissue and blood

To determinate the BBB integrity, we used the infusion of the Evans blue (EB) dye (113 mg/Kg), as shown previously [37]. Mice were sedated intraperitoneally with Diazepam (20 mg/Kg), and 200 μL of pyrogen-free saline (BioManguinhos, Fiocruz) containing EB dye (Sigma-Aldrich), administered via the orbital plexus. After 2 hours [37], mice were restrained physically and blood was collected by the orbital plexus, after anesthesia with the application of topical eye drops [38]. Exsanguination was practiced instead of perfusion, based on the efficiency of the technique [38] the shortest time needed per mouse, as we had a large number of animals per timepoint. Mice were euthanized at the end points (30, 60 and 90 dpi), using CO_2_ inhalation, followed by decapitation. According to a previously described protocol, the brains were collected through a craniotomy, weighted, rinsed by immersion in saline, photographed (Samsung Note 10), sagittally sectionized [38], and photographed again. Hemi-brain was placed in 1.5 mL Eppendorf tubes containing 500 μL of 10% formalin [38] for 10 days, to extract the EB. The eluate from each hemi-brain was collected and analyzed by spectrophotometry at 620nm [37, 38]. The concentration of the dye in each sample was determined using a standard curve, with serial dilution with the following concentrations: 3 μg/mL; 1 μg/mL; 0.3 μg/mL; 0.1 μg/mL; 0.03 μg/mL and 0 μg/mL (diluent). The final concentrations were calculated for whole brain.

### 2.11 Evaluation of cysts number and diameter

As described above, mice were euthanized using CO_2_ inhalation, followed by decapitation. Each brain collected was weighed, sagittally sectionized, and hemi-brain included in 1.5 mL of phosphate-buffered saline (PBS), then macerated initially using a 5ml syringe attached to a 18G hypodermic needle, making delicate movements (aspiration and disposal) until a homogenate is obtained. The process was repeated with a 21G hypodermic needle, to homogenize the smaller particles, until a homogenate was obtained. The number of cysts was determined by optical microscopy analyzing 20 μL of the homogenate in duplicate, and the total number calculated for the whole brain. The size of the cysts was determined using the digital software NIS Elements BR version 4.3 (Nikon Co., Japan), using images obtained with a Sight DS-U3 color vision digital camera adapted to an Eclipse Ci-S microscope. The diameter of the cysts was measured with the digital morphometric apparatus NIS Elements BR version 4.3 software (Nikon Co., Japan). Through a Sight DS-U3 color-view digital camera adapted to an Eclipse Ci-S microscope. The frequency distribution of the diameter length of the cysts was grouped into classes and the number of occurrences in each class was counted to know the behavior related to the size of the cysts, throughout the infection.

### 2.12 Histopathology

Encephala were collected, weighed, and sagittally cut, then fixed in 10% buffered formaldehyde in saline for 10 days, dehydrated and embedded in paraffin. Two sections of 4 to 6 μm thick sagittal sections were prepared and stained with hematoxylin and eosin. The slides were scanned with the Motic infinity 100 Scanner and viewed using the VM-Motic Digital Slide Assistance software, version 1.0.7.46. Histopathological changes and the distribution of cysts were analyzed, as well as the presence of silent cysts (devoid of surrounding inflammation). Radius between the two points determined the distance between each cyst and foci of inflammation. The cyst was considered silent when in a 10X field it was not possible to observe the presence of inflammation. Representative maps of the topographical location of the cysts in the brain were constructed using the stereotaxic coordinates of the mouse brain bregma (Fig 7A). The precision of the coordinates allows a potential error of less than 0.5 mm in the location of any point in the brain [39]. The cyst location was plotted using the Open-Source Scalable Vector Graphics Editor Inskscape software, version 1.0.1 (3bc2e813f5, 2020-09-07). Representative images were constructed overlaying the images of all analyzed mice.

### 2.13 Determination of cytokines in sera by CBA

The blood collected of EB-injected mice was used to obtain serum, stored in a -80°C freezer until CBA analysis. The levels of cytokines in sera were measured with the BD Cytometric Bead Array (CBA) Mouse Inflammation Kit (catalog 552364, BD Bioscience, USA). The kit was used for the simultaneous detection of interleukin 6 (IL-6), interleukin 10 (IL-10), interferon gamma (IFNγ), tumor necrosis factor (TNF), interleukin 12 (IL-12) and monocyte chemotactic protein (MCP-1/CCL2), in a single sample. The protocol was carried out according to the manufacturer’s recommendations. Cytokine standards were diluted serially to construct the calibration curves and used to determine the cytokines concentrations. The samples were analyzed using the 13-Color CytoFLEX-S flow cytometer (Beckman-Coulter, USA). Individual cytokine concentrations were indicated by their fluorescent intensities and expressed in pg/mL, using the FCAP Array Software. The theoretical limits of detection were: 5 pg/mL for IL-6, 2.5 pg/mL for IFNγ, 7.3 pg/mL for TNF, 10.7 pg/mL for IL-12 and 17.5 pg/mL for IL-10.

### 2.14 RT-PCR assay for detection of cytokine mRNA

Mice were anesthetized (300mg/Kg ketamine and 30mg/Kg of xylazine), blood was obtained by cardiac perfusion for 10 minutes with cold-saline and encephala were collected, at 20 and 45 dpi. RNA was isolated from CNS tissue of mice by acid guanidinium thiocyanate-phenol-chloroform extraction: RNA STAT-60^TM^. Reverse transcriptase-polymerase chain reaction conditions have been published elsewhere [40]. The PCR product and molecular weight marker were electrophoresed in a 6% polyacrylamide gel and stained with silver nitrate. Densitometry of gels was carried out on a Densitometer CS-9301PC (Shimadzu, Japan). The PCRs were standardized using hypoxanthine-guanine phosphoribosyl transferase (HPRT). Data are shown as relative IFNγ and TNF expression. Primers: **HPRT**: GTTGGATACAGGCCAGACTTTGTTG, GATTCAACTTGCGCTCATCTTAGGC, 30 cycles; **IFNγ**: AACGCTACACACTGCATCTTGG, GACTTCAAAGAGTCTGAGG, 32 cycles; **TNF**: GATCTCAAAGACAACCAACTAGTG, CTCCAGCTGGAAGACTCCTCCCAG, 28 cycles; **MIP-1α/CCL3**: CGCGGATCCCGGAAGATTCCACGCCAATTC, CGCGGATCCGGTTGAGGAACGTGTCCTGAAG, 32 cycles; **MIP-1β/CCL4**: CGCGGATCCCCCACTTCCTGCTGTTTCTCTTAC, CGCGGATCCAGCAGAGAAACAGCAATGGTGG, 33 cycles; **RANTES/CCL5**: CGCGGATCCCCACGTCAAGGAGTATTTCTACACC, CGCGGATCCCTGGTTTCTTGGGTTTGCTGTG, 26 cycles; **MCP-1/CCL2**: CCGGAATTCCACTCACCTGCTGCTACTCATTCAC, CCGGAATTCGGATTCACAGAGAGGGAAAAATGG, 30 cycles.

### 2.15 Statistical analysis

The sample size was determined based on the experience of our group and previous studies using the model of experimental toxoplasmic encephalitis; therefore, no formal sample size was calculated. To assess the normality of the data, the Kolmogorov-Smirnov and Shapiro-Wilks tests were used. To determine whether there were any significant statistical differences between the infected groups compared with the NI control groups, we applied the Student t-test with a 95% confidence level for data with normal distribution and the Mann Whitney test for data without normal distribution or ANOVA, when applicable. Correlation was analyzed using Pearson’s correlation coefficient. Statistical tests were performed using GraphPad Prism version 8.0. Differences were considered statistically significant when p < 0.05. The data were expressed as mean and standard error of the mean (SEM).

## 3. Results

### 3.1 Long-term chronic *Toxoplasma gondii* infection in C57BL/6 leads to loss of muscle strength with preservation of locomotor capacity

**Fig 2:**
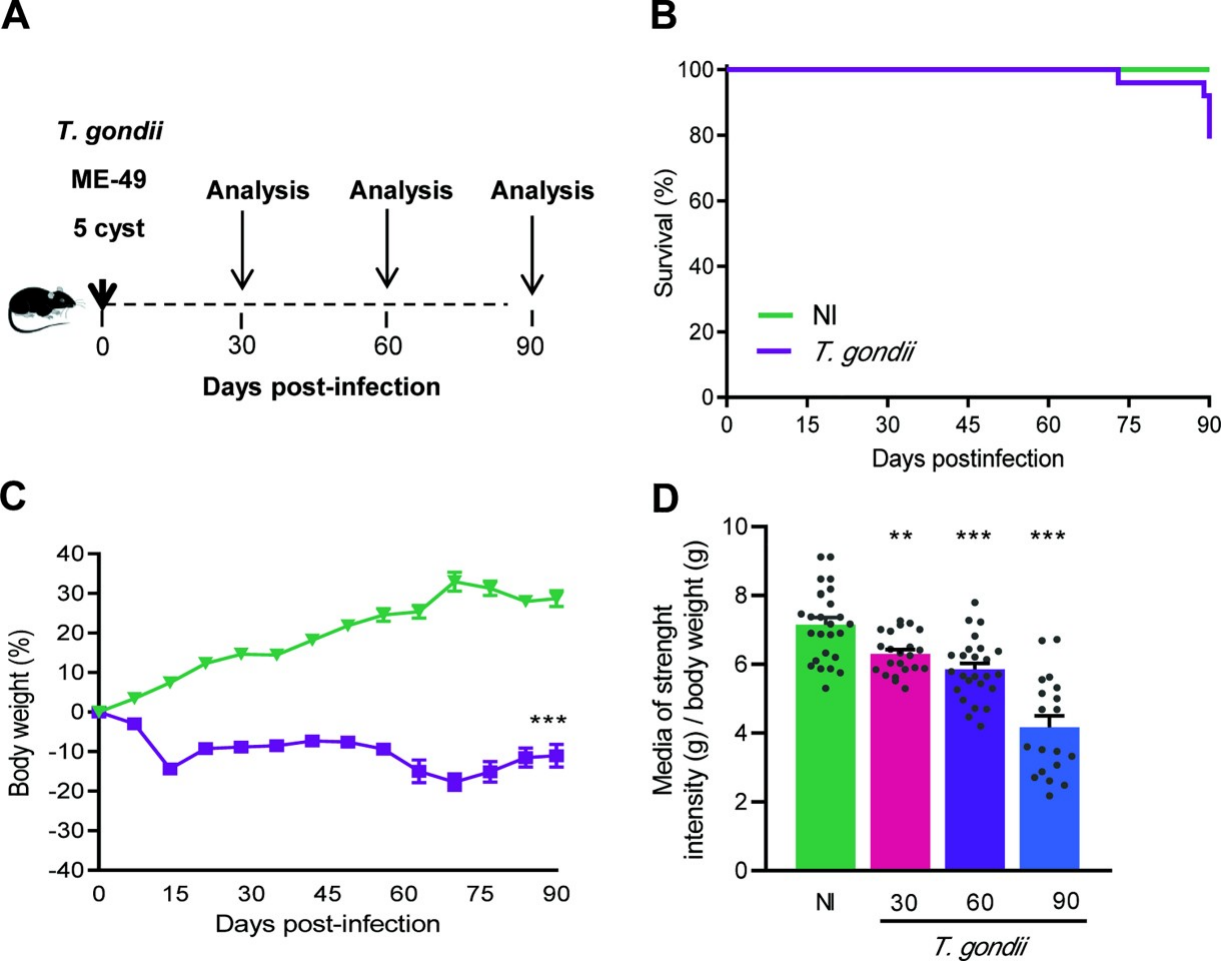
C57BL/6 mice chronically infected with the ME-49 *Toxoplasma gondii* strain survive and show weight and muscle strength loss. (**A**) Mice were infected with 5 cysts of the ME-49 *T*. *gondii* strain, the clinical follow-up and the mortality were assessed and recorded weekly, and the kinetics of infection was evaluated at 30, 60 and 90 dpi. (**B**) The survival curve shows a survival of 100% at 30 and 60 dpi, and 83% (19/24) at 90 dpi, compared to 100% in the age- and sex-matched NI control group. (**C**) Infected mice showed body weight loss during the acute phase of the infection (up to 15 dpi), after this period, the weight loss ceased. (**D**) Muscle strength was compromised in infected mice; values of muscle strength are shown as gram force (gf) / body weight (g). Each experimental group consisted of 4–6 NI mice and 10–19 *T*. *gondii-i*nfected mice. Each circle represents an individual mouse. Data are expressed as means ± SEM, and were analyzed using Welch’s test (**C**), and ordinary one-way ANOVA (**D**). **, *p*<0.01. ***, *p*<0.001, comparing *T*. *gondii-*infected and NI mice.

Female C57BL/6 mice were infected with 5 cysts of the ME-49 *T*. *gondii* strain. Kinetic of infection was evaluated at 30, 60 and 90 dpi (Fig 2A). The survival rate of infected mice was 100% up to 60 dpi, and 83% (5/24) survival was registered at 90 dpi (Fig 2B), compared to 100% in the NI control group. The analysis of all studied parameters showed no difference among the NI control groups run concurrently to *T*. *gondii*-infected mice, at 30, 60 and 90 dpi. Thus, for simplification, the collected data of all NI mice were gathered and referred as NI in graphs and figures. Body weight and clinical evolution were monitored weekly. The infected mice showed piloerection accompanied by body weight loss during the acute phase of the infection (up to 15 dpi). After this period, the weight loss ceased, nevertheless the body weight gain in infected mice remained lower than NI control mice (Fig 2C). In addition, the groups of infected mice showed loss of muscle strength at all timepoints of analysis (Fig 2D).

**Fig 3:**
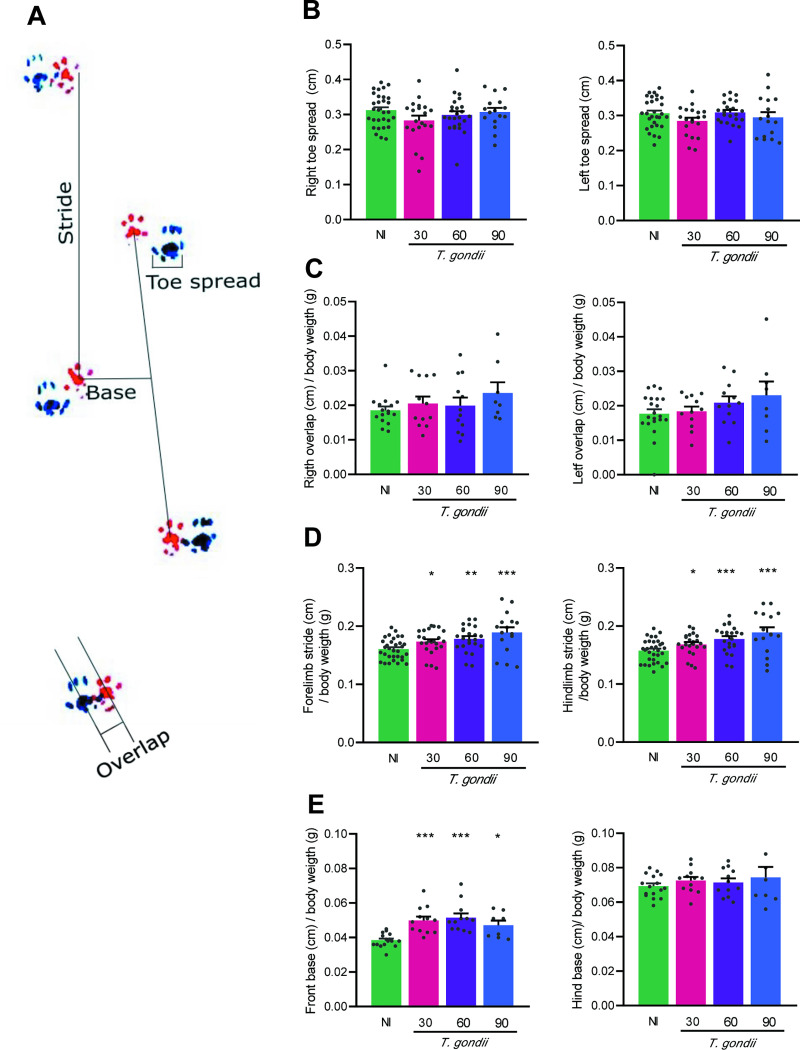
Mice with long-term chronic *Toxoplasma gondii* infection show preserved locomotor capacity. (**A**) The gait analysis was performed using the footprint test and were evaluated as such: the spread of the fingers and overlapping distance between the anterior and posterior limbs, right and left, and the stride length of limbs, width of the front and rear base. (**B**) The toe spread was normal in the right and left limb. (**C**) |No alteration was observed in the right and left step overlap of infected mice. (**D**) The forelimb and hindlimb stride were increased as the infection progresses. (**E**) The width of the front base was enlarged in infected mice, but the hind base was preserved. Each experimental group consisted of 4–6 NI mice and 8–12 *T*. *gondii-i*nfected mice. Each circle represents an individual mouse. Data are expressed as means ± SEM, and were analyzed using ordinary one-way ANOVA. *, *p*<0,05, **, *p*<0.01. ***, *p*<0.001, comparing *T*. *gondii-*infected and NI mice.

To explore whether the locomotor capacity was preserved, the gait pattern was also assessed using the footprint test (Fig 3A). At the three studied timepoints, no differences were detected in the width of the right and left toe spread (Fig 3B) and in the right and left step overlap (Fig 3C), when compared to NI mice. However, the forelimb and hindlimb stride were altered as the infection progresses (Fig 3D). Lastly, the width of the front base increased at 30, 60 and 90 dpi, contrasting with the preservation of the width of the hind base (Fig 3E). Therefore, the locomotor capacity of *T*. *gondii*-infected mice was preserved.

### 3.2 Long-term chronically *Toxoplasma gondii*-infected mice exhibit anxiety, depressive-like behavior, and hyperactivity

**Fig 4:**
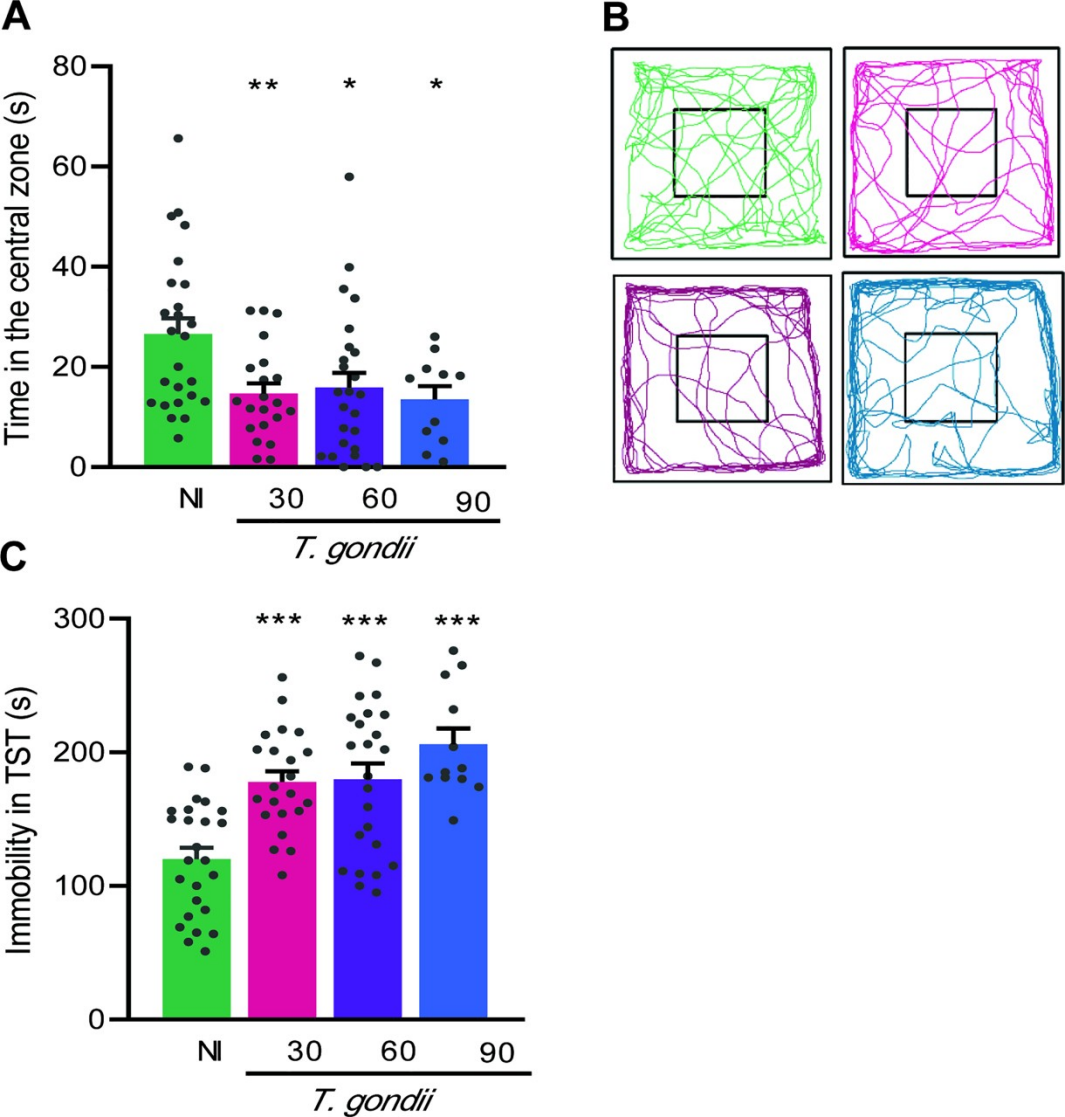
Anxiety and depressive-like behavior are detected in long-term chronically *Toxoplasma gondii*-infected C57BL/6 mice. (**A**) Chronically infected mice showed reduced time in the central zone in OFT, and (**B**) increased time exploring the peripheral area of the open field. (**C**) Depressive-like behavior was revealed as enhanced time of immobility in TST. Each experimental group consisted of 4–6 NI mice and 10–15 *T*. *gondii-i*nfected mice. Each circle represents an individual mouse. Data are expressed as means ± SEM, and were analyzed using *t-*Student test. *, *p*<0,05, **, *p*<0.01. ***, *p*<0.001, comparing *T*. *gondii-*infected and NI mice.

To assess anxiety, we used the OFT and analyzed the time expended in the central zone of the apparatus [29]. Our data showed that when compared to NI controls chronically infected mice exposed to the OFT remained reduced time in the central zone, at 30, 60 and 90 dpi (Fig 4A). More, increased time was expended in the peripheral area of the apparatus, revealed the intensity of the registered lines near the apparatus’ walls (Fig 4B). The presence of depressive-like behavior was evaluated using TST and revealed as enhanced time of immobility [31]. Consistently, when compared to NI controls chronically *T*. *gondii*-infected mice showed increased immobility time at early (30 dpi), and long-term (60 and 90 dpi) chronic infection (Fig 4C). Therefore, considering the standardized parameters in the early (30 dpi) and long-term (60 and 90 dpi) chronic *T*. *gondii*-infected mice showed depressive like-behavior and anxiety, compared with sex- and age-matched NI controls.

**Fig 5:**
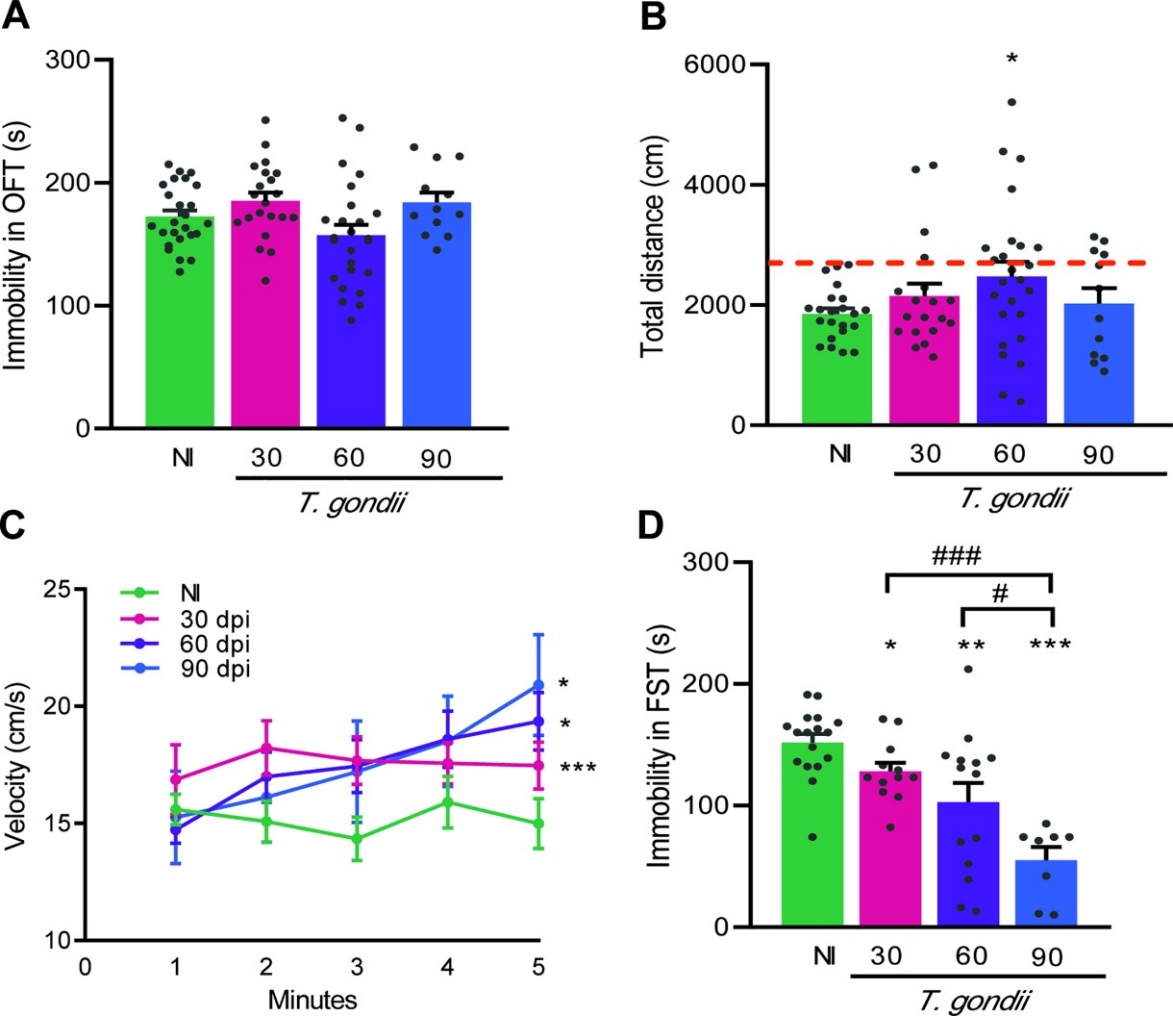
Hyperactive behavior is observed in chronically *Toxoplasma gondii*-infected C57BL/6 mice. To determine hyperactivity, we evaluated the time spent immobile, the distance and the speed of travel in OFT, and the activity in FST. (**A**) Infected mice did not show differences in immobility time when in the OFT. (**B**) At 60 dpi, mice showed increased distance traveled in OFT. At 30 (18%) and 90 (21%) dpi only a reduced percentage of mice showed increase in the distance traveled. Red dot line shows mean distance of NI group + 2 standard deviation. (**C**) The walk velocity in OFT was increased as infection progressed. (**D**) In FST, immobility time decreased as infection progressed. Each experimental group consisted of 4–6 NI mice and 8–15 *T*. *gondii*-infected mice. Each circle represents an individual mouse. Data are expressed as means ± SEM, and were analyzed using *t-*Student test (**A-C**) and the Mann-Whitney test (**D**). *, *p*<0.05, **, *p*<0.01. ***, *p*<0.001, comparing *T*. *gondii-*infected and NI mice.

Curiously, we observed that *T*. *gondii*-infected mice were apparently more active than NI mice. Thus, we refined our analysis and evaluated the time that infected mice spent immobile, and the distance and speed of travel inside the OFT. Initially, we did not notice differences in terms of immobility time in the OFT when chronically infected mice were compared to NI controls (Fig 5A). The analysis of the walked distance revealed that compared with NI controls 18%, 32% and 21% of infected mice traveled longer distances at 30, 60 and 90 dpi, respectively (Fig 5B). Indeed, the group of long-term chronically infected mice (at 60 dpi) walked longer distances than NI controls (Fig 5B). Moreover, compared with NI controls the walk speed was increased in early (30 dpi) and long-term (60 and 90 dpi) chronically infected mice (Fig 5C), thus suggesting aggravation of this change as the infection progresses. Altogether, these findings support an increase in the locomotor activity in chronically *T*. *gondii*-infected mice. Next, we tested the performance of infected mice in FST, a test to evaluate mice activity in adverse environments [33]. Compared to NI controls, we detected a significant decrease in the immobility time in all groups of infected mice submitted to FST. Further, this behavioral alteration is visibly aggravated with the course of infection, comparing 60 dpi with 90 dpi (*p* < 0.01) and 30 dpi with 90 dpi (*p* < 0.001) (Fig 5D). Altogether, increase in locomotor activity in OFT and reduced time of immobility in FST, also registered as increased time of activity, are suggestive of impulsive hyperactivity in early and, mainly, in long-term chronic *T*. *gondii* infection in C57BL/6 mice.

### 3.3 The number of cysts in the CNS decreases significantly throughout *Toxoplasma gondii* infection

**Fig 6:**
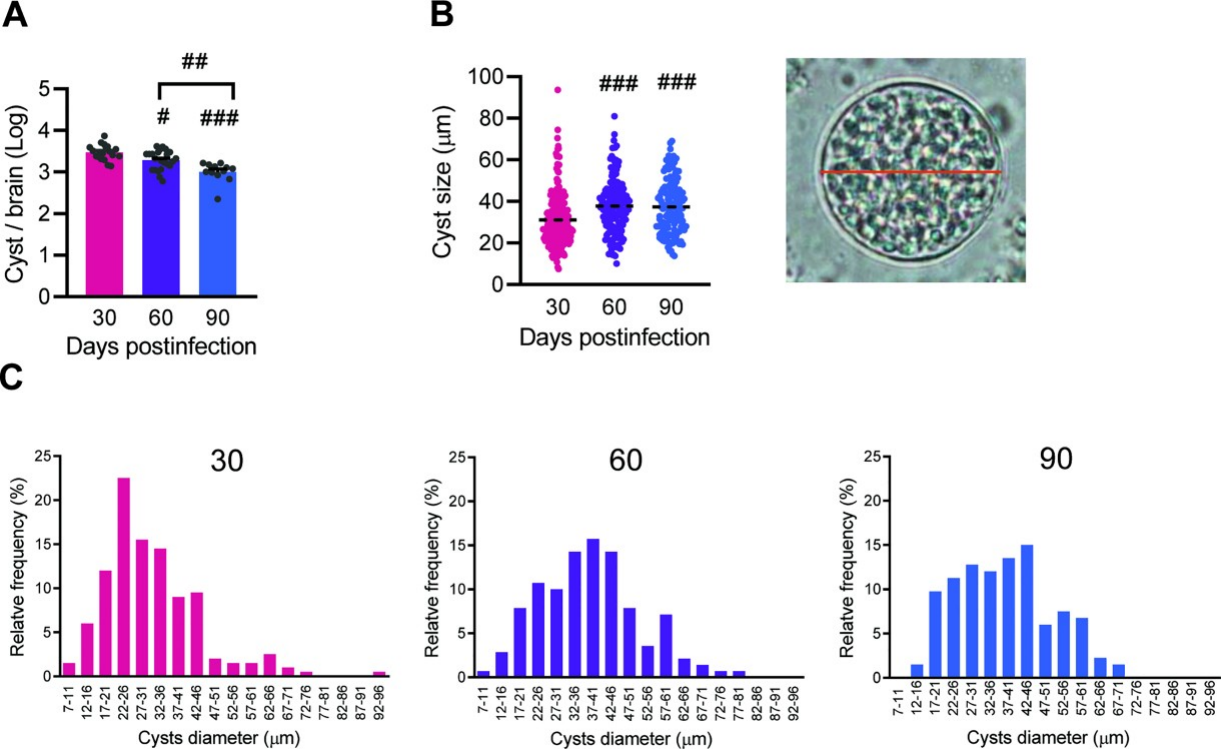
The numbers of parasite cysts in the brain decrease and the sizes increase as *Toxoplasma gondii* infection progresses in mice C57BL/6. (**A**) The number of cysts decreased as the early (30 dpi) infection coursed to long-term chronic infection (60 and 90 dpi). (**B**) The size of the cysts increased as infection progressed from early to long-term chronic phase. (**C**) The graphs show the relative frequencies of cysts in each class at the three timepoints analyzed. Each experimental group consisted of 8–12 *T*. *gondii*-infected mice. Each circle represents the number of cysts in the brain of each animal (**A**), and the diameter of each cyst (**B**). Data are expressed as means ± SEM, and analyzed using the Mann-Whitney test. #, p <0.05, ##, p <0.01. ###, p <0.001, comparing different timepoints.

Herein, the parasite load was determined by analyzing the number and size of *T*. *gondii* cysts present in the CNS of ME-49-infected mice. The number of cysts decreased as the early (30 dpi) chronic infection coursed to long-term chronic infection at 60 dpi (*p* < 0.05 compared with 30 dpi) and at 90 dpi (*p* < 0.001 vs 30 dpi; *p* < 0.01 vs 60 dpi), suggesting gradual control of infection (Fig 6A). However, the size of the cysts increased as infection progressed from early (30 dpi) to long-term (60 and 90 dpi) chronic phase (Fig 6B). Indeed, a more detailed analysis of the cysts size using the digital morphometric apparatus NIS Elements BR version 4.3 software (Nikon Co., Japan), revealed 18 size classes to be considered. Further, these data showed increase in the relative frequency of cysts > 42–46 μm in 60 and 90 dpi, compared with 30 dpi (Fig 6C).

Next, we tried to establish associations between the number of cysts in the CNS with the behavioral features analyzed, and no correlation was found in most of the analysis performed. Correlation (*p* < 0.05) was detected between the number of cysts in the CNS and immobility time in FTS S2A Fig. Further, correlation was observed between the number of cysts and the left and right forelimb strides (*p* < 0.05) and left and right hindlimb strides (*p* < 0.01) in the footprint test S2B and S2C Fig.

### 3.4 Cysts of *Toxoplasma gondii* prevailed in some regions of the encephalon

**Fig 7:**
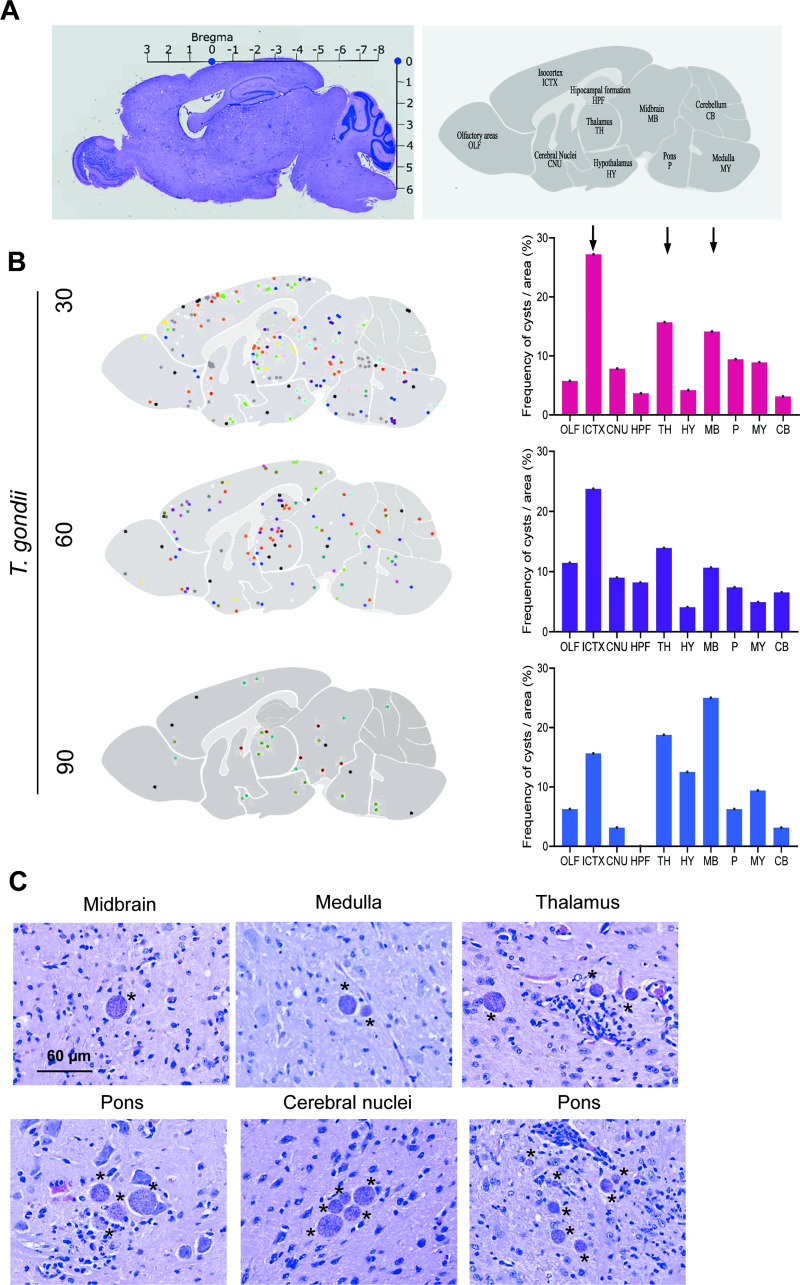
Some regions of the brain of C57BL/6 *Toxoplasma gondii*-infection mice are intensely infected. (**A**) The stereotaxic coordinates of the bregma of the mouse´s brain were used to construct representative maps of the topographic location of the cysts in each brain area: Olfactory areas (OLF), Isocortex (ICTX), Cerebral Nuclei (CNU), Hippocampal formation (HPF), Thalamus (TH), Hypothalamus (HY), Midbrain (MB), Pons (P), Medulla (MY) and Cerebellum (CB). (**B**) The cysts were localized in all brain regions, but the isocortex, thalamus and midbrain were colonized more intensely in the early (30 dpi) and long-term (60 and 90 dpi) chronic infection (each color represents an individual mice). The histograms show the percentage of cysts per area studied. (**C**) Representative pictures show individual cysts or multiple cysts per microscopic field in different brain areas, associated or not with inflammatory foci. Bar = 60 μm. Each experimental group consisted of 4–10 *T*. *gondii*-infected mice. Data were analyzed using ordinary one-way ANOVA.

Next, to evaluate a putative differential accumulation of *T*. *gondii* cysts in specific areas of the CNS, we analyzed two hematoxylin-eosin-stained histological sections per mouse. We divided the brain areas into: Olfactory areas (OLF), Isocortex (ICTX), Cerebral Nuclei (CNU), Hippocampal formation (HPF), Thalamus (TH), Hypothalamus (HY), Midbrain (MB), Pons (P), Medulla (MY) and Cerebellum (CB), according to Allen Institute for Brain Science [41], as shown in Fig 7A. At all timepoints evaluated (30, 60 and 90 dpi), the cysts were found in all areas of the CNS except for HPF at 90 dpi, when no cyst was observed in this area of any of the studied mouse. A larger number of cysts was detected in the ICTX, TH and MB areas, and this pattern was preserved in the three analyzed timepoints (Fig 7B and S3A Fig), supporting the existence of differential accumulation of *T*. *gondii* cysts in these CNS tissue areas. The size of the cysts does not vary due to the region in which they are located S3B Fig. Further, most cysts were observed isolated, but groups of 2 to 7 cysts were also found, regardless the studied areas of the brain (Fig 7C).

### 3.5 Behavioral changes are concomitant with generalized neuroinflammation in long-term chronic *Toxoplasma gondii* infection

**Fig 8:**
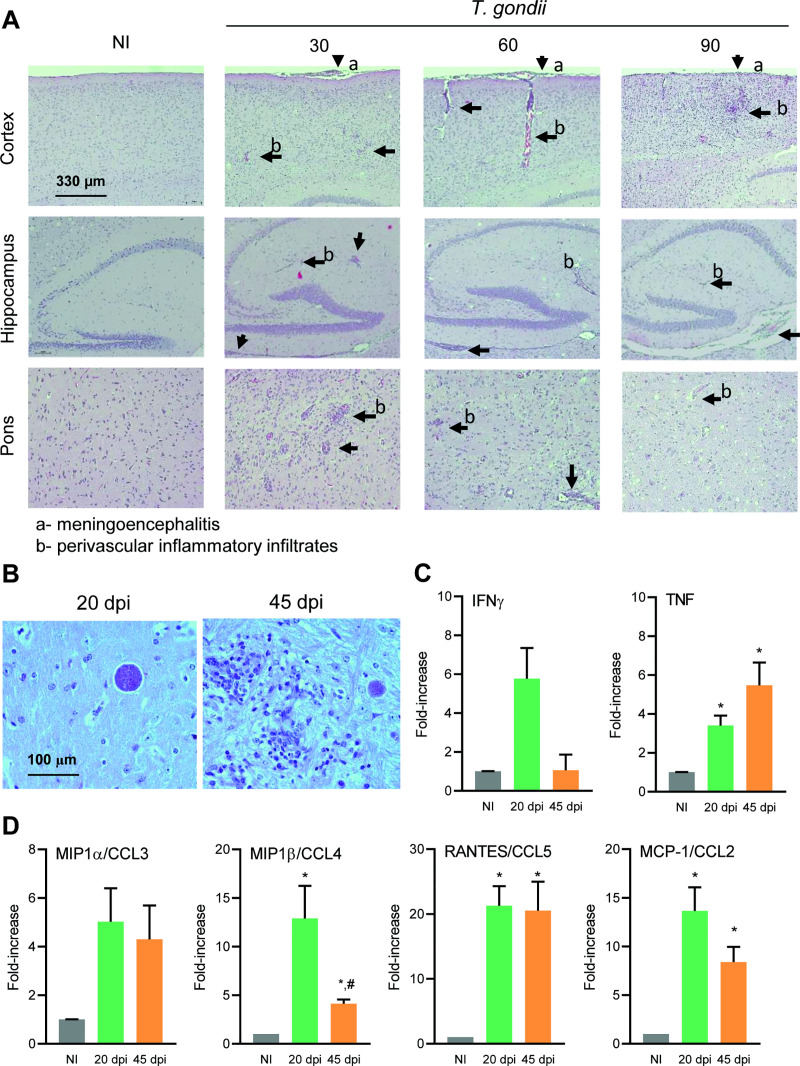
*Toxoplasma gondii*-infected C57BL/6 mice showed generalized neuroinflammation in early and long-term chronic phase of infection. (**A**) Infected mice presented inflammatory foci with meningoencephalitis and perivascular inflammatory cuffs in all evaluated areas of the CNS in early (30 dpi) and long-term (60 and 90 dpi) chronic infection. Bar = 330 μm. (**B**) Cysts devoid of inflammation were firstly detected in the CNS at the acute phase (20 dpi, left panel), and the inflammation is already settled in the chronic phase (45 dpi, right panel). Bar = 100 μm. (**C**) Increased expression of the pro-inflammatory cytokines IFNγ and TNF was detected in the acute phase, and TNF expression was sustained at the chronic phase of infection. (**D**) In the acute phase, the expression of MIP1α/CCL3, MIP1β/CCL4, RANTES/CCL5 and MCP-1/CCL2, was increased. The upregulation of the expression of TNF and CC-chemokines were sustained at 45 dpi. Each experimental group consisted of 2–4 NI mice and 2–10 *T*. *gondii*-infected mice, in two independent experiments. Data are expressed as means ± SEM, and were analyzed using ordinary one-way ANOVA. *, *p*<0,05 comparing *T*. *gondii-*infected and NI mice, and #, *p*<0.05 comparing acute and chronic groups of *T*. *gondii* infected mice.

In the three analyzed timepoints, infected mice presented inflammatory foci with meningoencephalitis and perivascular inflammatory cuffs composed of mononuclear inflammatory cells in all evaluated areas of the CNS (Fig 8A and S3C Fig). Rare apparently silent cysts devoid of inflammatory process were observed at 30 dpi (4.71%; 9/191) and at 60 dpi (4.09%; 5/122), while at 90 dpi no silent cyst was observed. Most of the cysts were surrounded or closer to inflammatory foci. A similar pattern was observed in the three evaluated timepoints in a way that the average distance between cysts and inflammatory foci ranged from 61.4 to 195.2 μm at 30 dpi, 104.9 to 182 μm at 60 dpi group, and 46.5 to 254.3 μm at 90 dpi S3C Fig. In addition, the association of cysts and inflammatory foci patterns was similar in different CNS areas.

Considering that mononuclear cells prevailed in neuroinflammatory processes at 30, 60 and 90 dpi, we settled an experiment to shed light on the pattern of cytokines and CC-chemokines driving putatively the migration of these cells. For that, a group of mice was analyzed at 20 dpi, when cysts devoid of inflammation were firstly detected in the CNS, and at 45 dpi, when inflammation was already settled (Fig 8B). Compared to NI controls, increased expression of proinflammatory cytokines IFNγ and TNF was detected at 20 dpi, therefore, preceding neuroinflammation. Further, TNF expression was sustained at 45 dpi (Fig 8C). At 20 dpi, the expression of the four CC-chemokines analyzed namely MIP1α/CCL3, MIP1β/CCL4, RANTES/CCL5 and MCP-1/CCL2, was enhanced, when compared to NI controls. Although at 45 dpi the expression of MIP1β/CCL4 and JE-MCP-1/CCL2 was reduced in comparison with the expression at 20 dpi, these CC-chemokines remained upregulated in this timepoint of chronic infection compared to NI controls (Fig 8D).

### 3.6 Systemic cytokine expression is upregulated at early and long-term chronic *Toxoplasma gondii* infection

**Fig 9:**
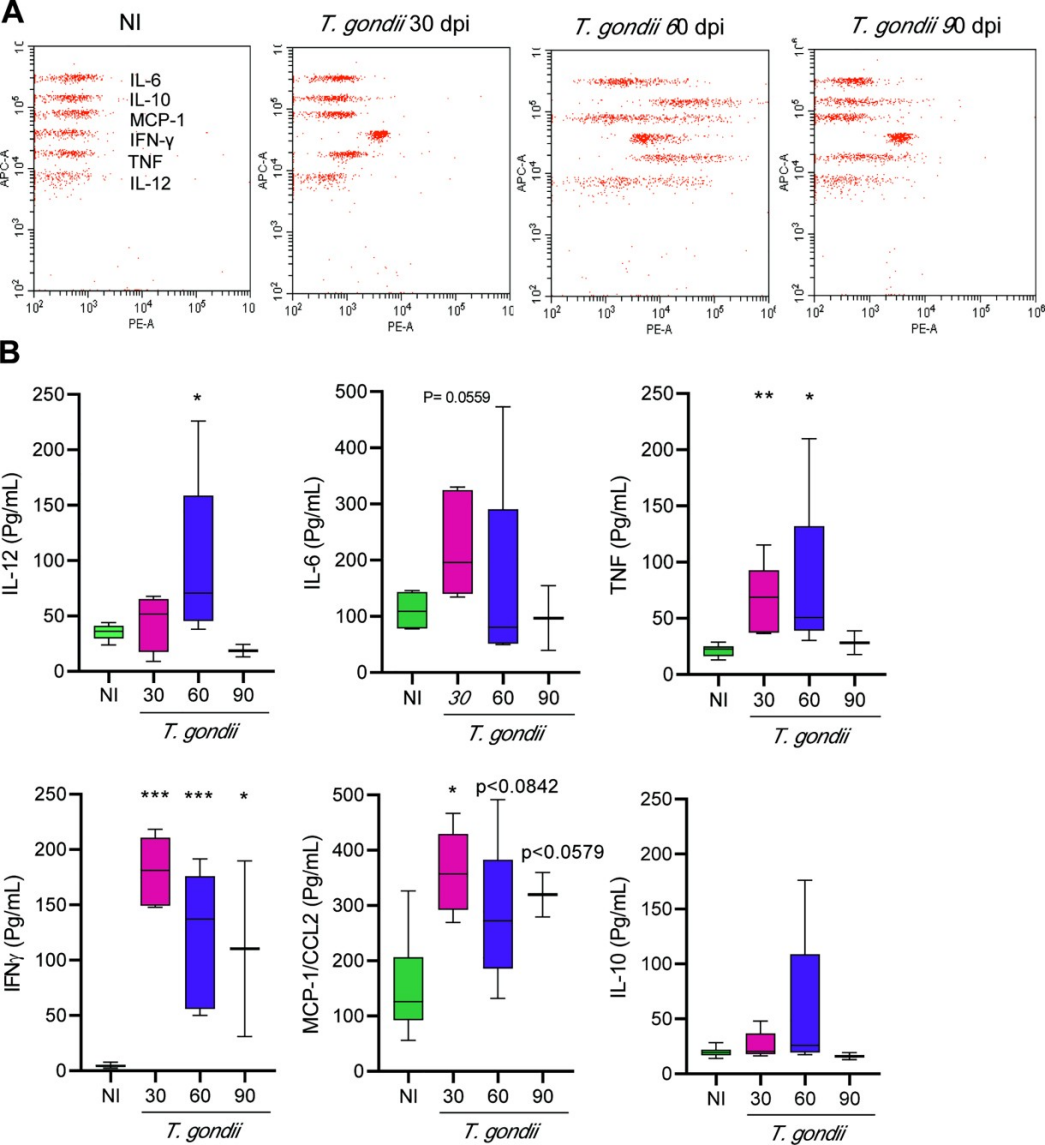
Systemic cytokine expression is upregulated in the early and long-term chronic *Toxoplasma gondii* infection. (**A**) The images show representative data plots of the FACS analysis of CBA. (**B**) Chronically *T*. *gondii*-infected mice showed increased levels of pro-inflammatory cytokines at 30 and 60 dpi. At 90 dpi, all cytokine levels showed a tendency to decrease or, even, exhibited cytokines levels like those found in sera of NI controls, except for IFNγ and MCP-1/CCL2 levels. Each experimental group consisted of 2–3 NI mice and 2–5 *T*. *gondii*-infected mice, in two independent experiments. Data are expressed as means ± SEM, and were analyzed using ordinary one-way ANOVA. *, *p*<0,05, **, *p*<0.01. ***, *p*<0.001, comparing *T*. *gondii-*infected and NI mice.

To assess systemic cytokine serum levels, blood of NI controls and *T*. *gondii*-infected mice was collected. The obtained sera were stored and submitted to simultaneous detection of cytokines, using the Mouse Inflammation CBA kit (IL-12, IL-6, TNF, IFN, MCP-1/CCL2, IL-10). Representative data plots of the FACS analysis are shown (Fig 9A), supporting that at 60 dpi all analyzed cytokines were upregulated, when compared to NI controls. In general, chronically *T*. *gondii*-infected mice increased serum levels of pro-inflammatory cytokines. The levels of TNF, IFNγ and MCP-1/CCL2 were elevated significantly at 30 and 60 dpi. At 90 dpi, levels of all cytokines showed a tendency to decrease or, even, cytokines levels were alike those found in sera of NI controls, except for IFNγ and MCP-1/CCL2 levels that remained upregulated at this timepoint (Fig 9B).

### 3.7 *Toxoplasma gondii* infection induces disruption of the blood-brain barrier and brain edema

**Fig 10:**
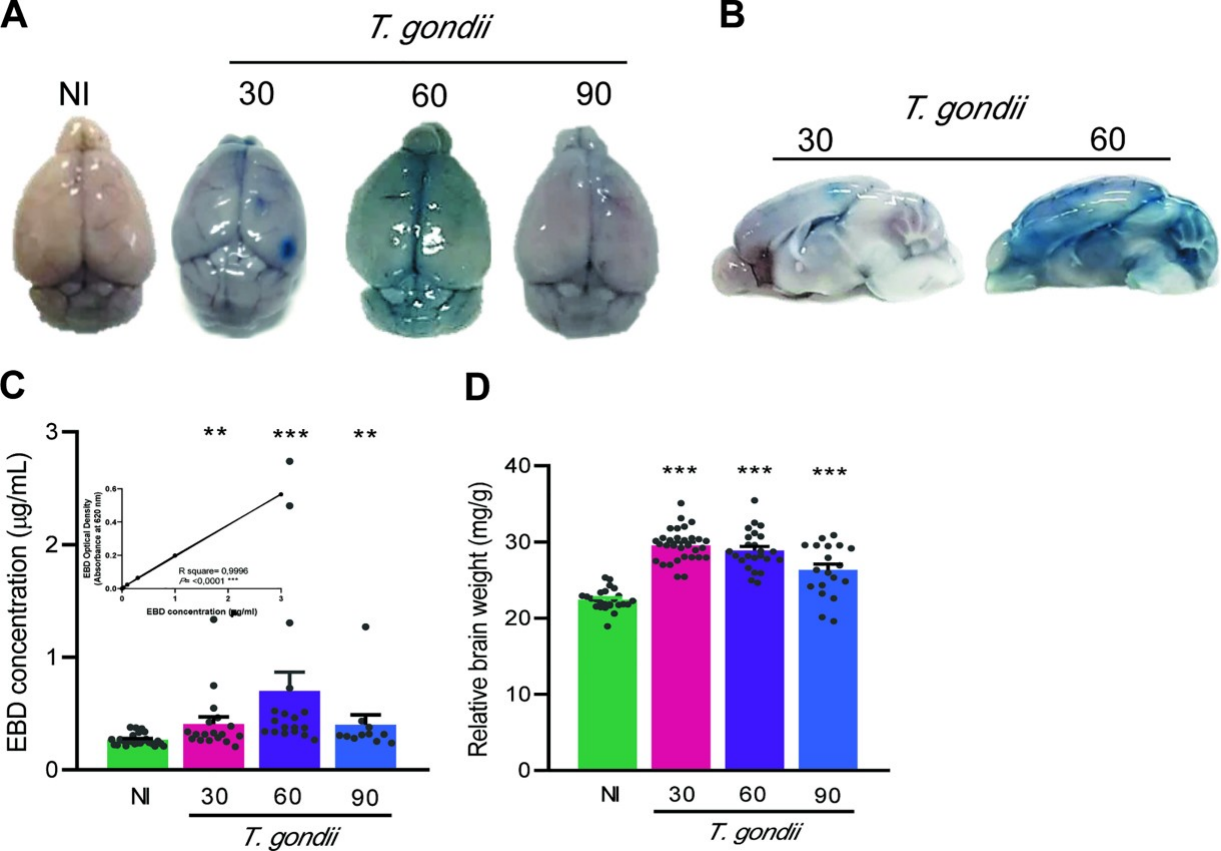
The increase in BBB permeability is concomitant with edema in *Toxoplasma gondii*-infected C57BL/6 mice. (**A**) Representative images of brains depicting EB extravasation at the timepoints assessed. (**B**) Representative sagittal sections of the encephala, at 30 and 60 dpi. (**C**) The concentrations of EB in the brain tissue of infected mice increased at the three studied timepoints, indicating the increase in BBB permeability. (**D**) Cerebral edema, manifested by a significant increase in the relative brain weight (brain weight in milligram / whole body weight in gram), was present in the timepoints evaluated. Each experimental group consisted of 4–6 NI mice and 8–19 *T*. *gondii-i*nfected mice, in two independent experiments. Each circle represents an individual mouse. The data are expressed as means ± SEM, and were analyzed using Mann-Whitey test (**C**) and *t-*Student test (**D**). **, *p*<0.01. ***, *p*<0.001, comparing *T*. *gondii-*infected and NI mice.

Based on the property of EB dye that binds proteins, mainly plasma albumin and on the physiological ability of the preserved BBB to be impervious to this protein, NI controls and infected mice were injected with EB and the encephala analyzed for EB extravasation, assumed as a biomarker of BBB disruption [42]. Representative images of brains depict localized and spotted EB extravasation at 30 dpi, while it is more evident in the whole brain at 60 dpi, and less noticeable at 90 dpi (Fig 10A). Representative sagittal sections of the encephala at 30 and 60 dpi corroborated this description (Fig 10B). When compared to NI controls, the quantitative data disclosed increased concentrations of EB in the encephala of infected mice at the three analyzed timepoints, with maximum levels achieved at 60 dpi, therefore, revealing BBB significant vascular permeability in *T*. *gondii*-infected (Fig 10C). Further, infected mice also presented a significant increase in relative brain weight in all evaluated timepoints (Fig 10D). Thus, associated with increased EB extravasation, these data are suggestive of cerebral edema.

## 4. Discussion

The present study evaluated the kinetics of the behavioral alterations of *T*. *gondii*-infected C57BL/6 mice, using a battery of standardized behavioral tests. Our data revealed that during the early (30 dpi) and long-term (60 and 90 dpi) chronic infection mice showed anxiety, depressive-like behavior and hyperactivity, concomitant with neuroinflammation, systemic inflammatory environment and BBB disruption. Further behavioral changes occurred regardless of the progressive decrease in the number of cysts and were independent of the localization of the cysts and inflammatory foci in the CNS areas. Thus, multiple factors may contribute to the observed behavioral abnormalities.

The experimental model we used, consisting of C57BL/6 mice infected with the ME-49 strain, allowed survival and chronic phase onset. Although our focus is on chronic infection, we monitored the clinical evolution of mice after the first day postinfection up to the end point. The mice showed a decline in body weight and the presence of piloerection in the first 15 days of infection, features proposed to be associated with the period of widespread parasite multiplication, typical of the acute *T*. *gondii* infection [43]. In the chronic phase, weight loss ceased, and body weight was steadied, alike previously described in other studies [9, 20, 44, 45]. Neuromuscular strength decreased in ME-49 strain-infected C57BL/6 mice, thus corroborating previous data that showed reduced neuromuscular strength in this mode [45] and in C57BL/6 mice infected with 10^3^ bradyzoites of ME49 strain [46]. Also, in chronically ME-49-infected Swiss Webster mice, decreased muscle strength was associated with myositis and reduction in muscle mass, being proposed that the inability to recover the weight during the chronic phase (> 30 dpi) is due, in part, to the continuous damage of the skeletal muscle [9, 45]. Likewise, muscle loss in the anterior tibialis, gastrocnemius and quadriceps muscles have been shown in chronically (37 dpi) ME-49-infected CBA/J mice [43]. Therefore, these data support that chronic infection characterized by cyst formation in the CNS, was associated with body weight loss, as reproduced in our experimental model.

Gait changes can represent a pathological state in many neurological diseases and disorders [47]. To analyze the locomotor capacity, the footprint test was performed, this is a gait analysis method, sensitive to neurodegenerative changes, and can be used in locomotor assessment of different disorders [48]. Here, we evaluated the spatial components of gait: stride length, width of the front and rear base, overlapping distance of the paw and spreading of the toes of right and left limbs. Stride length has been described as a reliable index of motor disorders caused by dysfunction of the basal ganglia in mice [49]. The increase in stance width generally indicates a compensatory gait pattern to avoid gait instability resulting from pain [50]. The overlapping distance of the limbs assesses the existence of greater flexibility of hip rotations or strength of the limbs to reach longer strides [47] as well as the uniformity of the alternation of steps [34] and, the toe spread can be used to form functionality indices for the sciatic, tibial, and peroneal nerves [50]. Here, the *T*. *gondii*-infected mice showed increase in the forelimb and hindlimb stride length at early and long-term chronic infection. The increase in stride length is influenced by the speed at which the mouse walks or runs, without affecting its gait [48, 49, 51]. The reduction in this parameter has been described in patients with neurodegenerative diseases such as Parkinson disease [52], and in murine models of Huntington disease [34, 53] and arthritis as a manifestation of pain [48, 54], but unbalanced gait was not observed in the model we described here. *T*. *gondii*-infected mice showed increase in the stance forelimb width. The increase in the width of the rear base, accompanied by a decrease in the length of the steps, was reported in a murine model of antigen-induced arthritis, maintained even after intra-articular treatment with AMPA/kainate glutamate receptors antagonist, which prevents pain and reduces pathology [55]. Thus, wider stance widths generally indicate a compensatory gait pattern used to protect a member of a painful movement and avoid gait instability [50]. In our infected mice, there were no signs of ataxia or foot drag detectable by the footprint test, and rear base width, toe spread and overlap of step were preserved. More, when compared to NI controls, the infected mice showed similar exploratory behavior, similar immobility time, and covered similar or longer distances in the OFT, besides the higher speed. So, we consider that these features support that our model maintains preserved locomotor capacity. In a previous study, chronically *T*. *gondii*-infected C57BL/6 mice with motor coordination deficits, impaired gait (mis-steps, stride length short, foot dragging in the rear paws), and reduced muscle strength, showed a reduction in exploration in the OFT, but no changes in social behavior or impaired cognitive function were observed, as object recognition test and spatial memory tests require displacement in the open field [46]. Interestingly, *Trypanosoma cruzi*-infected C57BL/6 mice show preserved muscle strength but alterations in cognitive function, also evaluated in tests that require displacement in the open field [25], supporting that these are dissociated features. Thus, our data suggest that decreased neuromuscular strength did not affect the motor function and the general locomotor activity of chronically ME-49-infected C57BL/6 mice.

Mice tend to explore a new environment, however anxiety behavior reduces time spent expended in central area and increases time consumed closer to the wall or peripheral area of the OFT apparatus [28]. The presence of anxiety, revealed as decreased time expended in the central area of the OFT, was detected in the early and long-term chronic infection of C57BL/6 mice with the ME-49 strain. Recently, similar results were shown in the model of early (30 dpi) chronic *T*. *gondii* infection of Swiss Webster mice [19]. In experimental models, TST and FST are widely used to assess depressive-like behavior in infectious and non-infectious diseases [24, 56–58]. It is expected that, when compared to their controls, afflicted mice subjected to both tests will increase immobility time as an indication of a depressive-like behavior [31, 33]. Here, in early (30 dpi) chronic *T*. *gondii* infection, mice showed an increase in the immobility time in TST, supporting a depressive-like behavior that is sustained as the infection progresses to the long-term chronic phase (60 and 90 dpi). The FSL rats, a lineage susceptible to behavioral alterations, showed depressive-like behavior when infected with the ME-49 *T*. *gondii* strain, and evaluated by sucrose preference test and FST [44]. Contrasting with the finding in FSL rats assessed in FST and added to our results of increased immobility in TST, we bring evidence that ME-49-infected C57BL/6 mice showed a decrease in the immobility time in FST, when compared to the NI controls. Thus, these conflicting results obtained in the two behavioral tests initially proposed to study depressive-like behavior applied to the same experimental groups, leading us to query the applicability of the FST to describe other behavior alterations. Truly, FST has been used previously to examine hyperactivity [59], stress coping behavior [60], while other authors suggest that the FST can be used to assess learning capability [61] and anxiety [62]. Discordant results have also been reported in non-infectious models, when no changes were exhibited in TST and reduced immobility time was shown in FST [57, 58]. Again, in a model of schizophrenia in Swiss Webster mice treated with a NMDA antagonist in the acute (single dose) or the chronic (10 consecutive days) process and submitted to FST and TST, the results were dissenting. In the acute protocol, increased immobility in TST was observed, and no changes were detected in FST. Conversely, in the chronic protocol immobility was increased in FST, whereas decreased immobility was detected in TST [56], supporting that these behavioral tests reveal different physiological mechanisms underlying the immobility process [56–58]. Chronic infection with *T*. *gondii* has been noticed as an anxiety reducer in mice exposed to elevated plus-maze test (EPMT) where they visited the open arms more frequently, even showing a decrease in the exploration of the central area of the OFT [10]. However, similar results in EPMT were also interpreted as changes in risk behavior, in model of chronic *T*. *gondii* infection [20]. We consider that like the FST, the results obtained in the EPMT may be misinterpreted, whenever EPMT allows to explore the impulsive behavior, one of the symptoms of attention-deficit/hyperactivity disorder (ADHD), which is expressed by the increase in exploratory activity in the open arms [63–67]. Likewise, we propose that the FST test can be a valid tool in the study of impulsivity in a murine model, though further studies are necessary. Anyhow, the reduced immobility in the FST, added to the increase in exploratory behavior and increased velocity in the OFT, led us to conclude that ME-49-infected C57BL/6 mice show a complex pattern of behavioral alterations with depressive-like behavior and hyperactivity in the early and long-term chronic infection, with aggravation of the process as the infection progresses. Interestingly, a recent study showed a relation between the severity of ADHD and IgG positivity for *T*. *gondii* infection in 6 to 18-year-old children, suggesting a role for the parasite infection in the exacerbation of ADHD [68]. Therefore, our results supporting hyperactivity in the OFT, depicted as increased velocity and total distance covered, corroborate the description of hyperactivity in the type II Prugniaud strain-infected BALB/cJ mice [22, 69], C57BL/6J infected with genetically modified ME-49 tachyzoites [20], and Swiss Webster infected with the ME-49 strain [19]. Notably, in our model regardless of the reduction in the number of brain cysts, the described behavioral changes were sustained at 60 and 90 dpi. In contrast, a relationship between the load of cysts and the severity of behavioral changes has been reported [10]. Indeed, our results revealed that the reduction in the number of brain cysts as infection progresses was not associated with reduction of hyperactivity, corroborating data in BALB/cJ mice chronically infected with the Prugniaud strain and treated with the antiparasitic guanabenz [22].

To shed light on possible biological contributors to the observed behavioral changes and knowing that the positioning of cysts in specific areas of the brain could give rise to certain behavioral changes [20], we investigated the topographical distribution of cysts and the proximity with inflammatory foci in the brain tissue. Since the cortex occupies 56% of the brain area [39], we divided it into different regions: olfactory areas, isocortex and hippocampal formation, to avoid bias in our results. Our model showed an accumulation of cysts in the isocortex, thalamus and midbrain in the three timepoints analyzed. The existence of tropism of *T*. *gondii* cysts to specific regions of the CNS is not entirely clear [70] and previous studies reported the heterogeneous distribution of cysts in the brain of mice. In C57BL/6 mice chronically infected with 30 cysts of the ME-49 strain, a larger infection of the hippocampus and telencephalon was observed, an area comprising the cerebral cortex and the cerebral nucleus [71]. In chronically ME-49-infected B6CBAF1/J mice, a heterogeneous distribution of brain cysts was observed, with enrichment of cysts in the frontal cortex and brain stem structures [10], as well as a higher parasite load in diencephalon, cortex and hippocampus in ME-49-infected Swiss Webster mice [9]. In Long-Evans rats infected with tachyzoites of the Prugniaud strain, a random distribution was observed throughout the forebrain, with enrichment in the preoptic area and the paraventricular hypothalamic nucleus [72]. CD1 mice infected with the HIF strain also showed an accumulation of cysts in the telencephalon [73]. These data led us to the support the existence of tropism of ME-49 strain to specific regions of the CNS, especially the cerebral cortex, and that tropism is dependent on the murine model and the *T*. *gondii* strain, indicating that further studies are required to explore this point. Cortical areas, limbic regions and basal ganglia are implicated in patients with depressive disorders [74], anxiety [75] and hyperactivity [76]. In addition, changes in the cerebellar brain region have been reported in patients with ADHD [76]. Despite the higher frequency of cysts in the isocortex, thalamus and midbrain showed in *T*. *gondii*- infected mice, the cysts were found to colonize all areas evaluated, although less frequently. Thus, it is not possible to link the location of the brain cysts detected in our model with the behavioral changes reported here. Once the influence of cysts´ localization in the brain on behavioral changes has been ruled out, we evaluated the influence of the size of ME-49 strain cysts, as cyst diameter, evaluating the kinetics of progression of the infection, and the influence of the size of the cyst in the affected brain area. We observed the presence of brain cysts of various sizes in all groups, indicative of an alternation of the growth and rupture phases underlying the population renewal of cysts [10], which did not allow correlation with behavioral changes. In addition, the size of the cysts by area of the brain was homogeneous, so it was not possible to observe any correlation. The variable observed in the three timepoints was neuroinflammation, detected in all brain regions, independently of the presence of cysts. Indeed, neuroinflammation was consistently described in previously analyzed experimental models of chronic toxoplasmosis [9, 10, 22]. Hyperactivity in a murine model of chronic *T*. *gondii*-infection was reduced with the use of guanabenz, a drug with potency to reduce neuroinflammation and perivascular cuff, but not the number of cysts in the CNS [22]. In addition, in ME-49-infected BALB/c mice, anxiety and short- and long-term memory impairment of new object recognition were reversed in mice treated with rosuvastatin. This therapy reduced the burden of tissue cysts in the brain and attenuated, but not resolve, the signs of neuroinflammation, including meningitis, perivascular cuffs, microglial proliferation, inflammatory cell infiltration and tissue damage [77]. The combination of sulfadiazine and pyrimethamine, the conventional therapy against toxoplasmosis [8], prevents the presence of parasites in the brain and the development of toxoplasmic encephalitis in murine models, but not the development of mild inflammatory lesions [78], which could still contribute to behavioral abnormalities, a question to be further explored. In humans, there are studies that demonstrate the efficacy of combined therapy in cognitive function in infants [79] and children [80] that developed normally the CNS and preserved intellectual function after treatment. However, the efficacy of this combined therapy regardng the cognitive functions of murine models of *T*. *gondii* infection is a matter to be further explored. In our study, mononuclear cells prevailed in the neuroinflammatory processes at 30, 60 and 90 dpi, thus we evaluated the profile of expression of cytokines and CC-chemokines, that could be involved in cell migration to the CNS, at 20 dpi (before neuroinflammation onset) and at 45 dpi (in the presence of neuroinflammation). We observed increased intracerebral expression of the proinflammatory cytokines IFNγ and TNF, and of the CC-chemokines, MIP1α/CCL3, MIP1β/CCL4, RANTES/CCL5 and MCP-1/CCL2 at 20 dpi, preceding neuroinflammation. At 45 dpi, when neuroinflammation is present, although the expression of IFNγ, MIP1β/CCL4 and MCP-1/CCL2 were reduced, the intracerebral expression of TNF and other CC-chemokines remained upregulated. Similar results were described in a model of toxoplasmic encephalitis in BALB/c mice, during the acute (10 dpi) and chronic (30 dpi) phases of the infection. In this model, increase in intracerebral mRNA of MIP1α/CCL3, MIP1β/CCL4, RANTES/CCL5 and MCP-1/CCL2 was dependent on IFNγ expression, as leukocyte recruitment to brain tissue was impaired in IFNγ-deficient mice [81]. Astrocytes, microglial cells and inflammatory leukocytes can produce intracerebral chemokines, that might act as facilitators of the recruitment, adherence and transendothelial migration of leukocytes through the BBB to the brain parenchyma, thus influencing the composition of the inflammatory infiltrate [81]. On the other hand, the peripheral inflammation can cause changes in cytokine levels in the brain through several mechanisms and, therefore, control inflammatory cell invasion of the CNS. Macrophages residing the CNS can be activated through the vascular endothelium [82] or through the circumventricular organs of brain regions devoid of BBB [83]. Also, circulating cytokines can be actively transported across the BBB [82], and may, therefore, activate glial cells to produce cytokines. Thus, leakage of cytokines of systemic plasma, together with the intracerebral cytokines and chemokines, could contribute to the maintenance of neuroinflammation. In our study, most of the assessed inflammatory cytokines (IL-12, IL-6, TNF, IFNγ and CCL2/MCP-1) showed high serum levels in the early chronic infection (at 30 dpi), with peak at 60 dpi and control at 90 dpi, except for IFNγ and CCL2/MCP-1, that persisted elevated. IL-10 serum levels, however, tended to increase only at 60 dpi. Thus, as the specific immune response is established, it may contribute to control parasite growth, leading to decrease the number of parasite cysts in the CNS, and contributing to reduce the stimulus to production of pro-inflammatory cytokines [84]. Previous kinetics study (7 to 70 dpi) in ME-49-infected mice has shown increased IL-12 and IFNγ levels in the acute phase with a decrease in the chronic phase of *T*. *gondii* infection, but without returning to baseline levels [85]. Molecules of *T*. *gondii* stimulate innate Toll-like receptors, which lead to the production of IL-12 that with TNF synergistically act to induce IFNγ production, as part of a robust Th1 immune response, crucial to establish an efficient antiparasitic response in the acute phase [86]. In addition, in chronic infection IFNγ is pivotal to control multiplication and dissemination of the parasite within the brain [81]. Indeed, depletion of this cytokine in the chronic phase leads to reactivation of the infection and inflammatory foci in the CNS [87]. Therefore, continuous systemic Th1 immune response, crucially IFNγ, is necessary to control *T*. *gondii* parasitism in the brain in the chronic phase of infection [84, 88]. IL-6 is traditionally described as a pro-inflammatory cytokine. However, it has been shown that increased IL-6 levels may regulate the production of IL-12 and IFNγ, resulting in an anti-inflammatory signal [89]. On the other hand, IL-6 plays a protective role in chronic infection, as IL-6-deficient ME-49-infected mice show high numbers of cyst and mortality, with severe toxoplasmic encephalitis with areas of necrosis [90]. IL-10 downregulates the expression of IL-12 and Th1 cytokines, but not the CC-chemokine CCL2/MCP-1 [91]. The anti-inflammatory response triggered by IL-10 may act favoring tissue repair but also contributing to the maintenance of cysts in the CNS [92]. The expression of the CC-chemokine CCL2/MCP-1 can be induced by inflammatory stimuli, such as TNF [93]. Moreover, intracerebral CCL2/MCP-1, acting via CCR2, plays a role in activating microbicidal mechanisms that control *T*. *gondii* parasitism in the CNS [93]. Altogether, our data suggest that the C57BL/6 mice model infected with the ME-49 strain triggered an efficient effector immune response, involving cytokines and CC-chemokines, that contributes to parasite control and reduction in the number of cysts in the CNS. Further, neuroinflammation, which may lead to behavioral changes, could be maintained through positive regulation of the intracerebral and peripheral pro-inflammatory cytokines and CC-chemokines, attracting inflammatory cells. A variety of neuroendocrine, neurochemical and behavioral changes are proposed to be consequence of peripheral immune activation, through the release of pro-inflammatory cytokines [11]. It has been shown that anxiety and depression can be induced by the systemic administration of IFNα in patients with hepatitis C [26]. Even so, mild peripheral inflammation in non-infected humans leads to impaired spatial memory [94]. In C57BL/6 mice infected with the ANKA *Plasmodium berghei* strain, which causes cerebral malaria, and cognitive deficits can be triggered by the early migration of mononuclear cells to the brain, facilitating the increase of chemokine levels in the brain [95]. The transient systemic inflammation induced by intraperitoneal administration of LPS to a mouse model of chronic neurodegenerative disease, can exacerbate inflammation in the CNS and accelerate disease progression as impaired motor coordination and cognitive function [96]. In absence of neuroinflammation, the role of TNF levels in serum in inducing depression-like behavior was demonstrated in a murine model of experimental chronic Chagas disease [24]. Thus, we suggest that the increased levels of cytokines and CC-chemokines in plasma and their leakage in the CNS may sustain neuroinflammation, which may result in the observed behavioral changes, anxiety, depression, and hyperactivity in chronic *T*. *gondii* infection.

The disruption of the BBB resulting from the establishment of a vascular or cerebral pathology will result in the leakage of serum-derived components to the CNS, which can lead to brain dysfunction affecting the thinking processes, mood and behavior, and generate psychiatric disorders [97]. A hypothesis has been proposed that the high rate of psychiatric diseases is associated with the breakdown of BBB thus assuming a relationship of peripheral inflammatory processes in psychiatric disorders [11]. In the last decades, data have sustained that disruption of the BBB allows the extravasation of pro-inflammatory cytokines and immune cells that can activate the CNS resident cells and, therefore, induce neurodegeneration, underpinning behavioral alteration [11]. Here, our data show that in the three timepoints BBB integrity was impaired and the relative brain weight was raised, suggestive of brain edema, supporting the leakage of blood-born molecules into the CNS putatively contributing to neuroinflammation. Similar data were obtained in chronically ME-49-infected Swiss Webster [98]. Inflammatory processes in the CNS may contribute to determine the severity and prognosis of neurological and cognitive disorders and can both cause and result from BBB dysfunction [99]. Truly, BBB impairment is associated with brain pathophysiology in several neurological disorders in humans and experimental mice models, including traumatic brain injury [100], stroke [101], epilepsy [102], autoimmune encephalitis, schizophrenia [103], Alzheimer disease [102] and depression [103, 104]. Similarly, psychiatric disorders are related to changes in levels of pro-inflammatory cytokines in serum [105–107]. Patients with bipolar disorder have elevated peripheral levels of IL-6 and TNF [105]. Increased IL-1β, IL-10 and TNF levels are present in patients with depression [106]. Likewise, IL-12, IL-6, TNF and IFNγ peripherical levels are enhanced in patients with schizophrenia [107], and IL-6 and IL-10 levels elevated in children with ADHD [108, 109]. In acute and chronic *T*. *cruzi* infection, C57BL/6 mice are refractory to neuroinflammation and upregulation of cytokine expression in the CNS but show depressive-like behavior. This behavioral change was associated with increased TNF levels in serum and abolished by anti-TNF antibody [24], reinforcing that in an infectious situation peripheral blood cytokine may overflow into the CNS and contribute to behavioral alterations.

Altogether, our data indicate that persistence of *T*. *gondii* cysts in the CNS may stimulate intracerebral cytokine and CC-chemokine production, contributing to recruit inflammatory cells, thus sustaining neuroinflammation and BBB disruption, which may allow the leakage of inflammatory mediators into the brain tissue. Hence, in chronic toxoplasmosis the systemic and brain-born inflammatory milieu may contribute to behavioral changes, as anxiety, depression, and hyperactivity (S1 Graphical abstract). Therefore, multifactorial components shall be considered when proposing a therapeutic approach to hamper progression or to reverse mental disorders associated with chronic *T*. *gondii* infection.

## Supporting information

S1 Checklist (PDF)

S1 Graphical abstract In the chronic phase of *T*. *gondii* infection, the persistence of parasite cysts in the brain may sustain neuroinflammation and BBB disruption, permitting leakage of serum cytokines into the CNS. The CNS inflammatory milieu may contribute to anxiety, depressive-like behavior, and hyperactivity.
(TIF)

S1 Fig Flow chart showing the experimental protocol with the number of animals used to assess the expression of intracerebral CC-chemokines and cytokines in non-infected (NI) and *T. gondii*-infected C57BL/6 mice, at 20 dpi and 45 dpi, in two independent experiments. (TIF)

S2 Fig Correlation between the number of cysts in the CNS with the behavioral features. (**A**) Correlation between the number of cysts and the immobility time in FTS. (**B**) Correlation between the number of cysts and the left and right forelimb stride in the footprint test. (**C**) Correlation between the number of cysts and the left and right hindlimb stride in the footprint test. Data were analyzed using Pearson’s correlation coefficient.
(TIF)

S3 Fig C57BL/6 mice showed generalized neuroinflammation with a greater presence of cysts in the isocortex, thalamus and midbrain, without influence of the size of the cysts. The analyses are shown the brain areas: Olfactory areas (OLF), Isocortex (ICTX), Cerebral Nuclei (CNU), Hippocampal formation (HPF), Thalamus (TH), Hypothalamus (HY), Midbrain (MB), Pons (P), Medulla (MY) and Cerebellum (CB). (**A**) The histograms show the total number of cysts for each area in the brain in the three analyzed timepoints. Large numbers of cyst were found in the isocortex, thalamus and midbrain areas. (**B**) The histograms show that the size of the cysts was not influenced by the brain region where they were localized. (**C**) The histograms show that most of the cysts were found surrounded or close to inflammatory foci, and a similar pattern was observed in the three evaluated timepoints. Each experimental group consisted of 4–10 *T*. *gondii*-infected mice. Data were analyzed using ordinary one-way ANOVA.
(TIF)
