# Supplementary figures and images for: Behavioral alterations in long-term Toxoplasma gondii infection of C57BL/6 mice are associated with neuroinflammation and disruption of the blood brain barrier
Source: PLoS One. 2021 Oct 5;16(10):e0258199. doi: 10.1371/journal.pone.0258199 (PMC8491889; doi:10.1371/journal.pone.0258199)

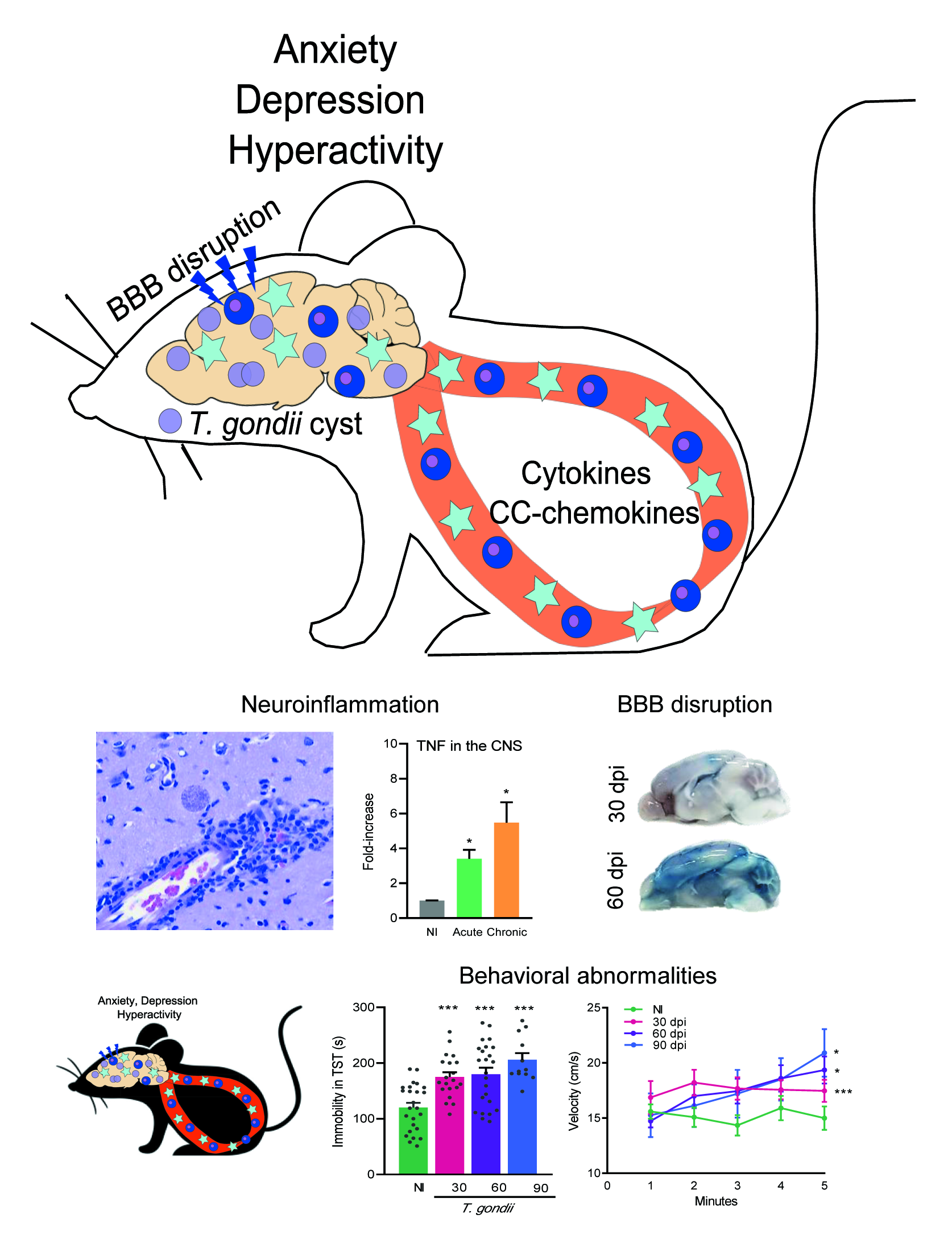

Supplement: S1 Graphical abstract — The CNS inflammatory milieu may contribute to anxiety, depressive-like behavior, and hyperactivity. (TIF) [file pone.0258199.s002.tif]

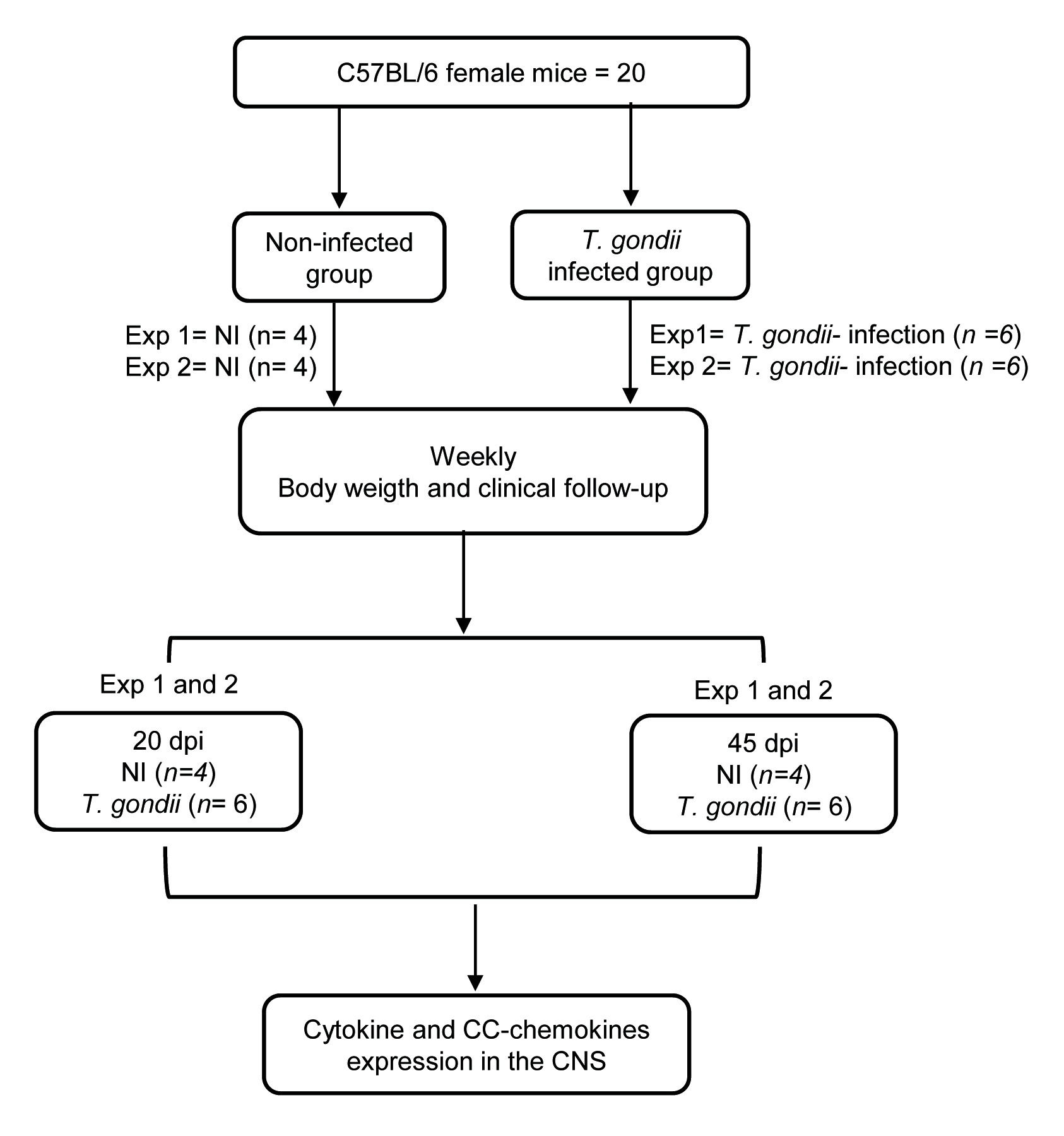

Supplement: S1 Fig — (TIF) [file pone.0258199.s003.tif]

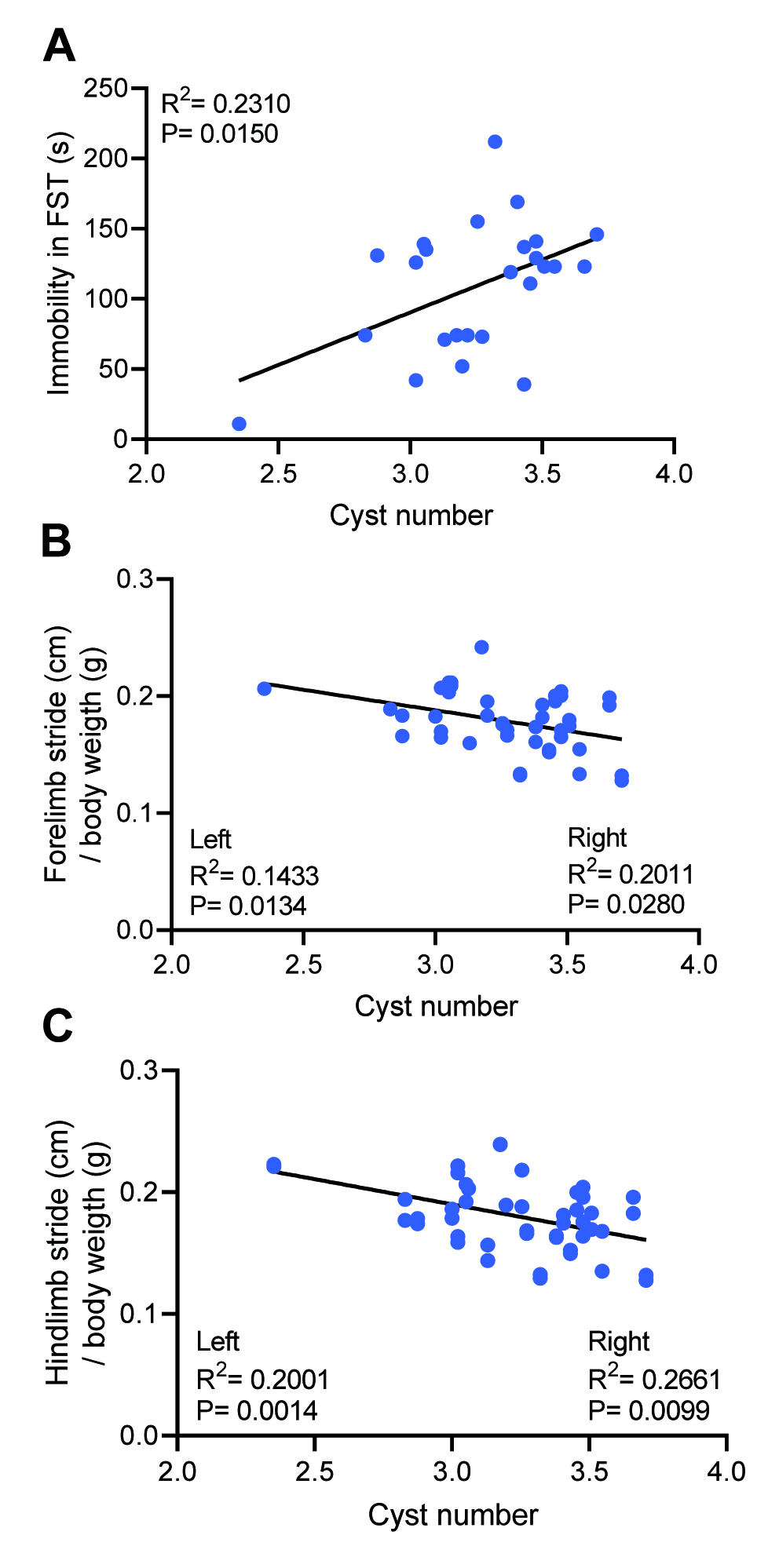

Supplement: S2 Fig — (A) Correlation between the number of cysts and the immobility time in FTS. (B) Correlation between the number of cysts and the left and right forelimb stride in the footprint test. (C) Correlation between the number of cysts and the left and right hindlimb stride in the footprint test. Data were analyzed using Pearson’s correlation coefficient. (TIF) [file pone.0258199.s004.tif]

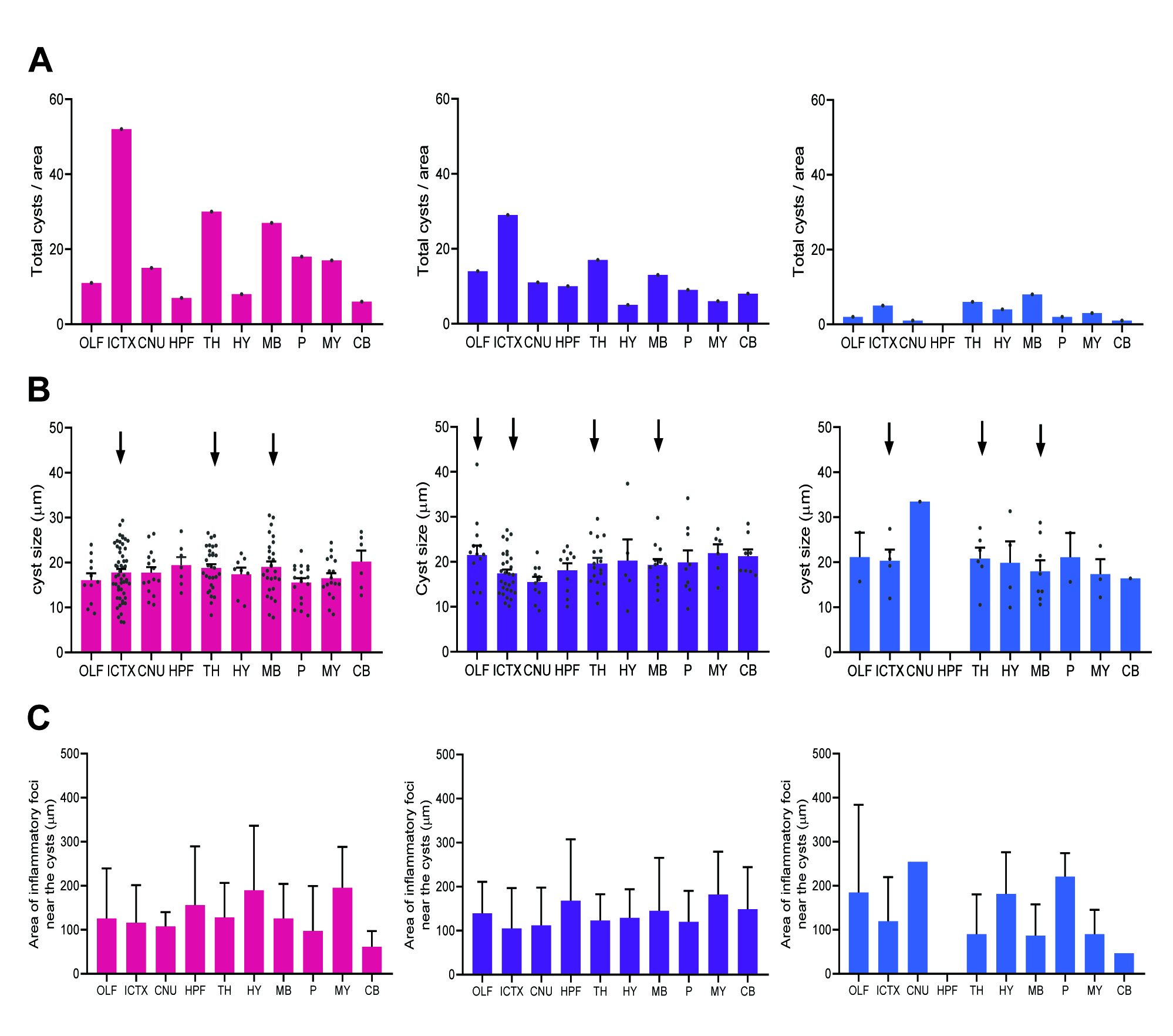

Supplement: S3 Fig — The analyses are shown the brain areas: Olfactory areas (OLF), Isocortex (ICTX), Cerebral Nuclei (CNU), Hippocampal formation (HPF), Thalamus (TH), Hypothalamus (HY), Midbrain (MB), Pons (P), Medulla (MY) and Cerebellum (CB). (A) The histograms show the total number of cysts for each area in the brain in the three analyzed timepoints. Large numbers of cyst were found in the isocortex, thalamus and midbrain areas. (B) The histograms show that the size of the cysts was not influenced by the brain region where they were localized. (C) The histograms show that most of the cysts were found surrounded or close to inflammatory foci, and a similar pattern was observed in the three evaluated timepoints. Each experimental group consisted of 4–10 T. gondii-infected mice. Data were analyzed using ordinary one-way ANOVA. (TIF) [file pone.0258199.s005.tif]
